# Chicoric acid-rich extract of *Cichorium intybus* seeds (CAREOCI) preserves pancreatic β-cell function under glucotoxic stress through modulation of MAFA, DYRK1A, cell cycle, apoptosis, and mitochondrial dysfunction

**DOI:** 10.3389/fphar.2026.1953632

**Published:** 2026-09-30

**Authors:** Anish Murtaja Alam Khan, Kailash Chandra, Furqan Choudhary, Aftab Alam, Farheen Azad, Jasmeet Kaur, Mohit Suyal, Yasmin Bano, Dinesh Kumar Singhal, Sayeed Ahmad, Bibhu Prasad Panda, Vineet Jain

**Affiliations:** 1 Hamdard Institute of Medical Sciences and Research (HIMSR), New Delhi, India; 2 Jamia Hamdard School of Chemical and Life Sciences, New Delhi, India; 3 Jamia Hamdard School of Pharmaceutical Education and Research, New Delhi, India; 4 ICMR - National Institute of Cancer Prevention and Research, Noida, India

**Keywords:** diabetes mellitus, *Cichorium intybus*, chicoric acid, pancreatic β-cell, glucotoxicity, DYRK1A, apoptosis, mitochondrial dysfunction

## Abstract

**Introduction:**

Progressive decline in functional pancreatic β-cell mass is a key pathological feature of type 2 diabetes mellitus (T2DM), highlighting the need for therapies that preserve and regenerate β-cells. This study investigated the preservative effects of a chicoric acid-rich aqueous extract of *Cichorium intybus* seeds (CAREOCI) under glucotoxic conditions (25 mM glucose) in pancreatic β-cells.

**Methods:**

Phytochemical standardisation by HPTLC, HPLC, and LC–MS/MS confirmed chicoric acid is the principal constituent present in CAREOCI. A molecular docking study identified DYRK1A is a one of the key target (−8.72 kcal/mol) of chicoric acid for β-cell regeneration. In glucotoxic MIN 6 β-cells, CAREOCI significantly enhanced glucose-stimulated insulin secretion, restored antioxidant status, reduced intracellular ROS, and suppressed IL-1β and TNF-α.

**Results:**

CAREOCI also reduced apoptosis, attenuated caspase-3/7 activity, preserved normal nuclear morphology, restored mitochondrial membrane potential, and promoted autophagy through increased acidic vesicular organelles. Cell-cycle analysis indicated a trend toward increased S-phase population, suggesting enhanced β-cell proliferation. At the molecular level, CAREOCI downregulated DYRK1A, NF-κB, mTOR, and CHOP while upregulating MAFA, PDX1, PAX6, and NKX6.1, supporting β-cell survival and preservation. Compared with purified chicoric acid, CAREOCI consistently exhibited greater efficacy, indicating potential complementarity among its phytoconstituents.

**Conclusion:**

CAREOCI protects pancreatic β-cells through coordinated antioxidant, anti-inflammatory, anti-apoptotic, mitochondrial-protective, autophagy-promoting effects, supporting beta cell preservation and function under glucotoxicity condition supporting its potential as a phytopharmaceutical for T2DM.

## 1 Introduction

Diabetes mellitus (DM) is a major global health challenge. In type 2 diabetes mellitus (T2DM), insulin resistance is a primary metabolic defect that increases the functional demand on pancreatic β-cells, while persistent glucotoxicity, lipotoxicity, oxidative stress, mitochondrial dysfunction, and endoplasmic reticulum (ER) stress progressively impair β-cell function and survival (Diane et al., 2024; Dinić et al., 2022). Excessive reactive oxygen species (ROS) and ER stress activate mediators such as C/EBP homologous protein (CHOP/DDIT3), promoting β-cell dysfunction and apoptosis (Oyadomari and Mori, 2004). β-cell preservation and regeneration are distinct: preservation maintains the viability and function of existing β-cells, whereas regeneration involves restoration or expansion of β-cell mass through proliferation or replacement (Aguayo-Mazzucato and Bonner-Weir, 2018; Dor et al., 2004). Adult β-cell proliferative capacity is inherently limited, particularly under conditions of chronic metabolic stress. Therefore, promoting β-cell regeneration while preserving residual β-cell function remains a significant therapeutic challenge.

DYRK1A is an important regulator of β-cell proliferation through the NFAT pathway. DYRK1A-mediated phosphorylation restricts NFAT nuclear signalling, whereas DYRK1A inhibition promotes NFAT-dependent β-cell proliferation (Dirice et al., 2016; Wang et al., 2015). Hence, DYRK1A represents a mechanistically relevant target for investigating strategies aimed at maintaining or restoring β-cell mass. mTOR plays a central role in nutrient sensing, cellular growth, proliferation, and the maintenance of β-cell mass, whereas CHOP is a key mediator of ER stress–induced β-cell injury (Balcazar et al., 2009; Gu et al., 2011; Song et al., 2008). Together, these pathways provide a mechanistic framework linking β-cell proliferation, growth, and cellular stress.

Regeneration of pancreatic β-cells requires not only restoration of cell mass but also the preservation of β-cell identity and functional integrity. Key transcription factors, including PDX1, NKX6.1, PAX6, and MAFA, play essential roles in maintaining β-cell identity, insulin gene expression, and mature β-cell function (Hang and Stein, 2011; Matsuoka et al., 2004). In addition, mitochondrial homeostasis and autophagy are critical for β-cell survival and function, as disruption of these can exacerbate oxidative and ER stress, thereby contributing to β-cell dysfunction and injury (Ebato et al., 2008; Quan et al., 2012). The coordinated actions of MAFA and other key β-cell transcription factors are essential for maintaining β-cell function, and targeting these interconnected regulatory pathways may provide a complementary strategy for preserving β-cell integrity under metabolic stress.

*Cichorium intybus* contains bioactive compounds including chicoric acid, chlorogenic acid, quercetin, and rutin and has demonstrated glucose-lowering, antioxidant, and insulin-sensitizing effects (Azay-Milhau et al., 2013; Ferrare et al., 2018; Nasimi Doost Azgomi et al., 2021). Our previous studies demonstrated beneficial metabolic and antioxidant effects of *Cichorium intybus* extracts. Chicoric acid, one of the characteristic phenolic constituents of *Cichorium* intybus, has also been investigated for its direct metabolic effects. Previous studies have shown that chicoric acid can enhance glucose uptake and stimulate insulin secretion in pancreatic islets, suggesting that its antidiabetic effects extend beyond systemic glucose lowering and may involve direct effects on pancreatic endocrine function (Tousch et al., 2008). However, whether a chicoric-acid-rich *C. intybus* preparation directly influences β-cell proliferation, identity, mitochondrial function, ER stress, apoptosis, and autophagy under glucotoxic conditions remains poorly understood. Therefore, the present study investigated the preserving effects of CAREOCI on glucotoxicity-induced pancreatic β-cell dysfunction. We examined the effects of CAREOCI on expression of MAFA and other transcription factors, DYRK1A-associated signaling, cell-cycle progression, apoptosis, and mitochondrial dysfunction to elucidate its potential mechanisms of β-cell preservation.

## 2 Materials and methods

*Cichorium intybus* (chicory) seeds were procured from a local vendor and taxonomically authenticated by a qualified taxonomist.

### 2.1 Extract preparation and standardization

*Cichorium intybus* seeds were cleaned, shade-dried for 4–5 days, and crushed into a coarse powder. The powdered material was soaked in distilled water at a 1:10 (w/v) ratio for 12 h at room temperature. The soaked material was subsequently heated in a boiling-water bath until the volume was reduced to approximately half of the initial volume. The suspension was filtered, and the resulting filtrate was concentrated by evaporation to obtain the crude aqueous extract. The concentrated extract was dried, weighed to determine the extraction yield, and stored in airtight containers at 4 °C until further use.

#### 2.1.1 Phytochemical analysis (HPTLC, HPLC and LC-MS)

Phytochemical standardization of the chicoric acid-rich extract of *C. intybus* seeds (CAREOCI) was performed using HPTLC, HPLC, and LC-MS/MS following previously reported methods (Bahri et al., 2012). HPTLC fingerprinting was performed on Merck HPTLC silica gel 60 F_254_ plates (200 × 100 mm) using a CAMAG Linomat five applicator and CAMAG TLC Scanner 4 controlled by visionCATS software. Chicoric acid standard (TCI, Japan) was prepared in HPLC-grade methanol at concentrations of 62.5, 125, 250, 500 and 1,000 μg/mL. Samples were applied as bands using an application speed of 150 nL/s with a 0.20 µL pre-dose volume. The first track position was set at 20.0 mm, with an inter-track distance of 11.4 mm. Chromatographic development was performed in a TTC 20 × 10 developing chamber using ethyl acetate: formic acid: acetic acid: water (30:1.5:1.5:7, v/v/v/v) as the mobile phase. The chamber was saturated for 20 min, and the plates were developed to a solvent-front distance of 70 mm. Following development, the plates were dried at room temperature for 5 min and scanned in absorbance mode at 254 and 366 nm using the CAMAG TLC Scanner 4. Chicoric acid in extract was identified by comparison with the reference based on its chromatographic profile and an Rf value of approximately 0.70. Densitometric profiles were recorded using VisionCATS software.

HPLC analysis was performed following the method reported by Bahri et al. (Bahri et al., 2012), with minor modifications. Briefly, separation was achieved on a C18 column using gradient elution with 1% acidified water (solvent A) and acetonitrile (solvent B) at a flow rate of 0.5 mL/min, with detection at 320 nm. Chicoric acid was identified by comparison with an authentic reference standard based on its retention time (approximately 32 min) and subsequently quantified in CAREOCI. Further phytochemical characterization was performed using LC-ESI(+)-QTOF-MS. Metabolites were tentatively identified based on accurate mass, retention time, fragmentation patterns, and comparison with published literature and spectral databases.

### 2.2 *In silico* analysis

Target proteins associated with pancreatic β-cell proliferation, identity, survival, and stress responses were selected for molecular docking analysis. These included DYRK1A (dual-specificity tyrosine-phosphorylation-regulated kinase 1A (PDB ID: 3ANQ), mTOR (mechanistic target of rapamycin (PDB ID: 8ERA), NF-κB (nuclear factor kappa B (PDB ID: 4G3D), PAX6 (paired box 6 (PDB ID: PAX6), PDX1 (pancreatic and duodenal homeobox 1 (PDB ID: 2H1K), MAFA (V-Maf musculoaponeurotic fibrosarcoma oncogene homolog A (PDB ID: 4EOT), NKX6.1 (NK6 homeobox 1 (PDB ID: P78426), and CHOP/DDIT3 (CCAAT/enhancer-binding protein homologous protein (PDB ID: P35638). These proteins/targets were selected based on their roles in β-cell proliferation, maintenance of β-cell identity, survival, insulin secretion, and cellular stress responses (Blandino-Rosano et al., 2012; Dirice et al., 2016; Guo et al., 2013; Hang and Stein, 2011; Meng et al., 2012; Oyadomari and Mori, 2004; Schaffer et al., 2013). Molecular docking was performed using Schrödinger Maestro to investigate the predicted interactions of chicoric acid with the selected targets, and molecular interactions were visualized using PyMOL and Discovery Studio. The three-dimensional structures of the target proteins were obtained from the RCSB Protein Data Bank. For DYRK1A, the crystal structure 3ANQ (chain A) was used; missing residues were remodelled using the AlphaFold server with 3ANQ chain A as the structural reference, followed by structure refinement using the Protein Preparation Wizard in Schrödinger Maestro, including correction of structural inconsistencies, assignment of appropriate protonation states, and energy minimization. For molecular docking analysis, receptor grids of 20 × 20 × 20 Å were generated around the selected binding-site residues for all the target proteins investigated in this study, including MAFA, PAX6, PDX1, DYRK1A, NF-κB, mTOR, NKX6.1, and CHOP. For DYRK1A, the grid was centered on the selected binding-site residues Val173, Ala186, Lys188, Glu203, Phe238, Leu241, Leu294, and Val306. Chicoric acid (PubChem CID: 5281764) was retrieved from PubChem and prepared using the LigPrep module to generate an optimized ligand structure and relevant protonation/tautomeric states. The prepared ligand was docked into the generated receptor grid using the selected Schrödinger docking protocol, and the resulting poses were ranked according to their docking scores. Chicoric acid showed a predicted docking score of −8.72 kcal/mol against DYRK1A, indicating a favourable predicted interaction with the target binding site.

### 2.3 Cell culture study

The MIN 6 mouse pancreatic β-cell line was used as an *in vitro* model. MIN 6 cells were procured from the National Centre for Cell Science (NCCS), Pune, India, and all experiments were performed using cells at passage number 55–57. Cells were routinely maintained in Dulbecco’s Modified Eagle Medium (DMEM; HiMedia, AL007S) containing 4.5 g/L glucose, 1 mM sodium pyruvate, L-glutamine, and 1.5 g/L sodium bicarbonate, supplemented with 10% fetal bovine serum (FBS; HiMedia, RM10434). Cells were cultured at 37 °C in a humidified atmosphere containing 5% CO_2_ and were subcultured upon reaching 80%–90% confluence. The cells were confirmed to be negative for *mycoplasma* contamination and were subsequently used for all experimental assays. For further experiments, cells were seeded at assay-specific densities according to the respective plate and allowed to stabilize under standard culture conditions before treatment (O’Driscoll et al., 2004).

#### 2.3.1 Cytotoxicity assay

The cytotoxicity of CAREOCI was evaluated using the MTT colorimetric assay following the previously reported method of Kaur et al. with minor modifications. MIN 6 cells were seeded in 96-well plates at 1 × 10^4^ and allowed to stabilize for 24 h under standard culture conditions. Cells were subsequently treated with CAREOCI at concentrations of 10, 200, 400, 600, 800, and 1,000 μg/mL for 24 h. Following treatment, MTT was added at a final concentration of 0.5 mg/mL and cells were incubated for 4 h to allow the formation of formazan crystals. The culture medium was then carefully removed, and 100 µL DMSO was added to each well to dissolve the formazan crystals. Absorbance was measured at 570 nm using a microplate reader.

#### 2.3.2 Dose selection for subsequent *in-vitro* experiments

CAREOCI dose selection was guided by the traditional use of *C. intybus* seeds (5–7 g/day) (M Goi. (2016), The Unani Pharmacopoeia of India Part-II Volume-III), our previous preclinical and clinical studies in T2DM (Chandra et al., 2018; 2020), extraction yield, and preliminary MTT findings. With an 8.2% (w/w) extraction yield, 7 g of crude seeds corresponds to approximately 574 mg extract/day (Shazia Parveen et al., 2019). Based on the above and the observed cell-viability profile, 120 μg/mL CAREOCI was selected for subsequent *in-vitro* experiments. Further, 100 μg/mL purified chicoric acid was included as a comparator to determine whether the observed effects of CAREOCI were attributable primarily to chicoric acid or to the combined effects of its phytochemical constituents (Tousch et al., 2025).

#### 2.3.3 Glucotoxicity model of β-cell dysfunction

MIN 6 cells were seeded at 2 × 10^5^ cells/well in 6-well plates for glucotoxicity assays. Cells were divided into experimental groups as follows: Control 1 (normal glucose), maintained under basal glucose conditions (4.5 g/L glucose) and further treated with DMSO; Control 2 (glucotoxicity control), exposed to additional 25 mM D-glucose for 24 h and further treated with DMSO; and the CAREOCI-treated group, exposed to additional 25 mM D-glucose for 24 h, followed by treatment with CAREOCI (120 μg/mL) for an additional 24 h. For both the glucotoxicity control and CAREOCI-treated groups, 25 mM D-glucose was added to the basal glucose concentration already present in the culture medium (Hwang et al., 2021).

#### 2.3.4 Effect of CAREOCI on oxidative stress, antioxidant, and inflammatory markers

The effects of CAREOCI on oxidative stress, antioxidant, and inflammatory markers were evaluated in MIN 6 cells following 25 mM glucose-induced glucotoxicity and after 24 h treatment of CAREOCI and Chicoric acid. SOD activity, nitric oxide (NO), and total antioxidant capacity (TAC) were measured using the pyrogallol auto-oxidation method, Griess reaction (Damame et al., 2021), and a commercial assay kit (Abbkine Scientific Co), respectively. Cellular antioxidant capacity and lipid peroxidation were further assessed using EZAssay™ CUPRAC and TBARS kits (CellBio/HiMedia Laboratories Pvt. Ltd., India), while TNF-α and IL-1β levels were quantified using sandwich ELISA kits (BT Laboratory, Shanghai, China) according to the manufacturers’ instructions.

#### 2.3.5 Intracellular reactive oxygen species (ROS) measurement

After 24 h of glucotoxicity induction and treatment, intracellular ROS levels were assessed using 10 μM DCFDA (TargetMol Chemicals Inc., United States). Cells were incubated with DCFDA at 37 °C for 30 min in the dark. Subsequently, fluorescence was measured using a CytoFLEX flow cytometer (Beckman Coulter, United States) in the FITC channel (Ex/Em: 488/530 nm), as previously described by (Damame et al., 2021).

### 2.4 Effect of CAREOCI on the expression of molecular targets associated with β-cell dysfunction

Total RNA was extracted from MIN 6 pancreatic β-cells using TRIzol™ Reagent (Thermo Fisher Scientific, United States) according to the manufacturer’s instructions (Rio et al., 2010). The extracted RNA was reverse-transcribed into cDNA using the cDNA Synthesis Kit (G-Biosciences, India). Gene expression analysis was performed by quantitative real-time PCR using 2× PCR Master Mix (G-Biosciences, United States) and gene-specific primers according to the manufacturers’ protocols. The sequences of the gene-specific primers used in the study are presented in Supplementary Table S3. Relative mRNA expression levels were normalized to β-actin (ACTB) as the reference gene, and relative gene expression was calculated using the 2^−ΔΔCt^ method, with the respective control group and assigned a relative expression value of 1.0. Values > 1 and <1 represented increased and decreased expression, respectively, relative to the control gene expression (Livak and Schmittgen, 2001).

### 2.5 Effect of CAREOCI on cell cycle progression

Cell-cycle distribution was analysed by flow cytometry following propidium iodide (PI) staining. After glucotoxicity induction and 24 h treatment with CAREOCI, MIN 6 cells were harvested, stained with PI (50 μg/mL), and analysed using a CytoFLEX flow cytometer (Beckman Coulter, United States). The percentages of cells in the G_0_/G_1_, S, and G_2_/M phases were quantified to evaluate treatment-induced changes in cell-cycle progression.

### 2.6 Effect of CAREOCI on apoptosis

#### 2.6.1 Apoptosis assessment by annexin V-fitc/PI assay

Apoptosis was evaluated using the Annexin V-FITC/PI Apoptosis Detection Kit (Elabscience Biotechnology Inc., United States) according to the manufacturer’s instructions. MIN 6 cells were seeded at 5 × 10^5^ cells/well in 6-well plates in triplicate and allowed to stabilize under standard culture conditions. Cells were then exposed to 25 mM D-glucose for 24 h to induce glucotoxicity, followed by treatment with CAREOCI (120 μg/mL) for 24 h. Following treatment, cells were harvested, washed with PBS, and resuspended in binding buffer. Cells were stained with Annexin V-FITC and propidium iodide (PI) according to the manufacturer’s recommended concentrations and incubation conditions. Samples were analyzed using a CytoFLEX flow cytometer (Beckman Coulter, United States). At least 10,000 events were acquired per sample, and data were analyzed using CytExpert software to determine the percentages of viable, early apoptotic, late apoptotic, and necrotic cells.

#### 2.6.2 Effect of CAREOCI on caspase-3/7 activity

Caspase-3/7 activity was assessed using the Caspase-Glo® 3/7 Assay (Promega Corporation, United States) according to the manufacturer’s instructions. MIN 6 cells were seeded at 5 × 10^4^ cells/well in 24-well plates in triplicate and allowed to stabilize. Cells were exposed to 25 mM D-glucose for 24 h, followed by treatment with CAREOCI (120 μg/mL) for 24 h. Following treatment, Caspase-Glo® 3/7 reagent was added according to the manufacturer’s recommended volume and incubated for 1 h at room temperature in the dark. Luminescence was measured using a microplate luminometer. Caspase-3/7 activity was expressed as relative luminescence units (RLU) after background subtraction. All experiments were performed in triplicate.

#### 2.6.3 DAPI nuclear morphology assay

Following treatment, MIN 6 cells were fixed with 4% paraformaldehyde and stained with DAPI (1 μg/mL; SRL Scientific, India) for 15 min at 37 °C. Nuclear morphology was subsequently examined using a Leica DMRE confocal microscope equipped with a TCS-SPE confocal scanning head (Leica Microsystems, Wetzlar, Germany), with the assessment of nuclear morphological changes performed as described previously by Zakłos-Szyda et al., (Zakłos-Szyda et al., 2020).

### 2.7 Effect of CAREOCI on mitochondrial function, and autophagic activity

#### 2.7.1 Mitochondrial membrane potential (ΔΨm) analysis

Mitochondrial membrane potential (ΔΨm) was assessed using Rhodamine 123 (10 μg/mL; Thermo Fisher Scientific). Following treatment, cells were incubated with the dye for 30 min at 37 °C in the dark, washed with PBS, and imaged using a Leica DMRE confocal microscope (Leica Microsystems, Wetzlar, Germany). Fluorescence intensity was quantified using ImageJ software following background correction and expressed relative to the respective control groups, with Control 1 representing the normal-glucose condition and Control 2 representing the 25 mM glucose condition (Damame et al., 2021).

#### 2.7.2 Autophagy assessment by acridine orange staining

Autophagic activity was evaluated by Acridine Orange (AO; 1 μg/mL) staining. Following treatment, cells were incubated with AO for 15 min at 37 °C in the dark, washed with PBS, and imaged using a Leica DMRE confocal microscope (Leica Microsystems, Wetzlar, Germany). Green and red fluorescence images were merged to visualise acidic vesicular organelles (AVOs), and fluorescence intensity was quantified using ImageJ after background correction (Wu et al., 2022).

### 2.8 Statistical analysis

All experiments were performed in triplicate (n = 3), and the results are presented as mean ± standard deviation (SD). Statistical analyses were performed using GraphPad Prism version 8.0. Comparisons among multiple groups were analyzed using one-way analysis of variance (ANOVA) followed by Tukey’s multiple comparison test. For experiments involving two independent variables, two-way ANOVA followed by Tukey’s *post hoc* test was employed. A value of p < 0.05 was considered statistically significant.

## 3 Results

### 3.1 Quality analysis of chicoric acid rich extract of *cichorium intybus* seeds (CAREOCI)

Aqueous extraction of *C. intybus* seeds yielded 2.56 g of dried extract, corresponding to an extraction yield of 8.2%. The obtained aqueous extract was subsequently processed for phytochemical analysis. Based on its chicoric acid content, the standardized extract was designated as chicoric-acid-rich extract of *C. intybus* (CAREOCI).

#### 3.1.1 Phytochemical analysis of CAREOCI

The phytochemical fingerprint of CAREOCI was evaluated by HPTLC to assess the presence of chicoric acid. The CAREOCI extract exhibited eleven distinct chromatographic peaks, indicating a characteristic phytochemical profile. A prominent peak at Rf ≈ 0.70 in the CAREOCI chromatogram corresponded to the chicoric acid standard at the same Rf value, supporting the presence of chicoric acid in CAREOCI (Figure 1). Chicoric acid was further quantified by HPLC using a calibration curve ranging from 62.5 to 1,000 μg/mL, which showed good linearity (y = 447,18x − 379,287; *R* ^2^ = 0.9972) (Supplementary Figure S1). The chicoric acid concentration in CAREOCI was 173.6 μg/mL. LC–ESI(+)-QTOF-MS analysis revealed diverse phytochemical constituents, including alkaloids, hydroxycinnamic acids, phenolic acids, flavonoids, glycosides, and lipid-related metabolites. Chicoric acid was tentatively assigned at a retention time of 6.8251 min with an observed m/z of 475.3204 ([M + H]^+^). Other constituents were tentatively assigned based on accurate mass, retention time, fragmentation patterns, and comparison with available literature and databases. The corresponding MSI identification levels are provided in Supplementary Table S1.

**FIGURE 1:**
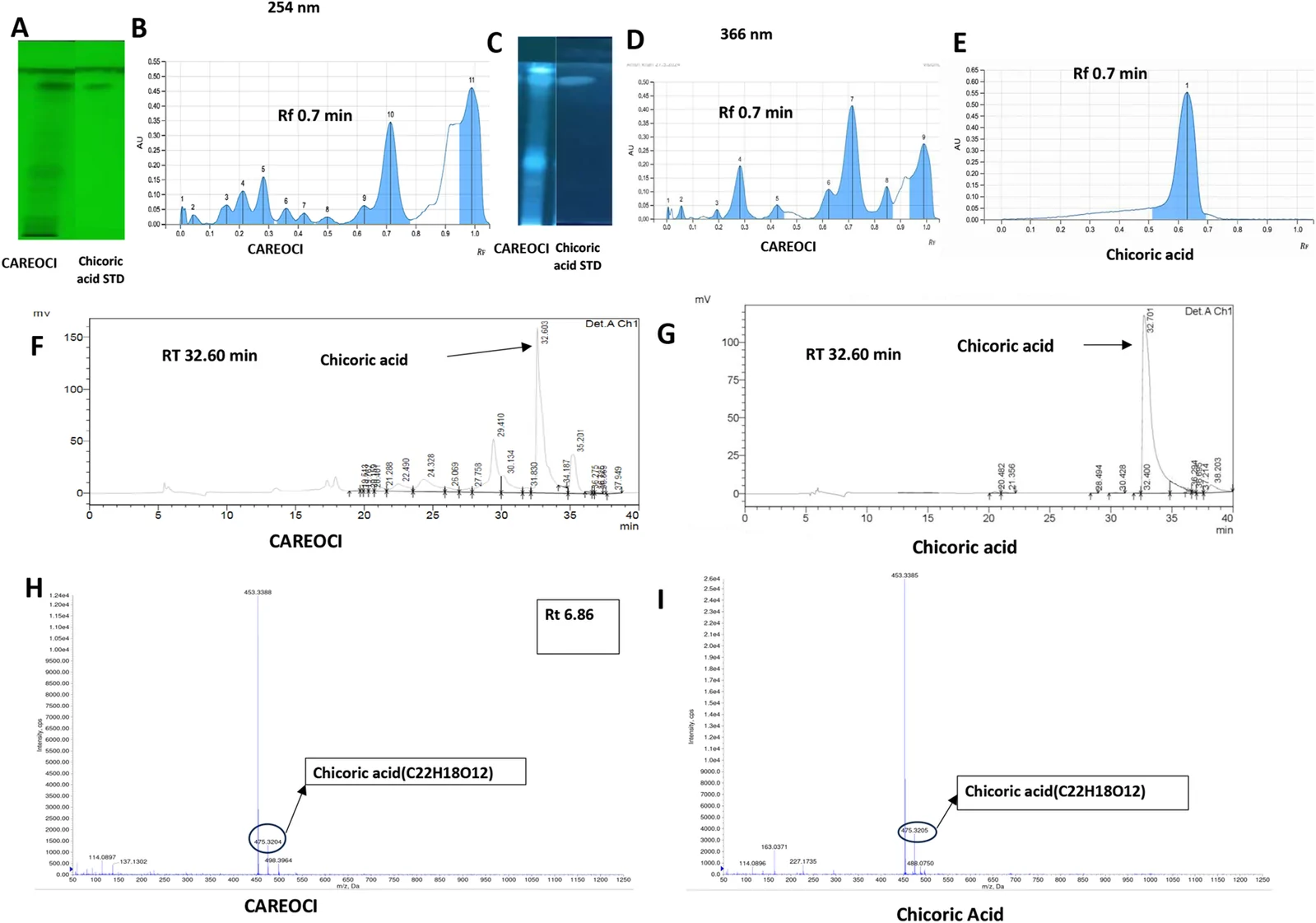
Phytochemical standardization of CAREOCI using HPTLC, HPLC, and LC–MS analysis. **(A)** HPTLC plate of CAREOCI and 1 mg/mL chicoric acid standard observed at 254 nm. **(B)** HPTLC densitogram of CAREOCI at 254 nm showing multiple chromatographic peaks, including a prominent peak at Rf ≈ 0.70 corresponding to chicoric acid. **(C)** HPTLC plate of CAREOCI and 1 mg/mL chicoric acid standard observed at 366 nm. **(D)** HPTLC densitogram of CAREOCI at 366 nm showing a characteristic peak at Rf ≈ 0.70. **(E)** HPTLC densitogram of the 1 mg/mL chicoric acid standard showing a characteristic peak at Rf ≈ 0.70. **(F)** HPLC chromatogram of CAREOCI showing a major peak corresponding to chicoric acid at a retention time (RT) of approximately 32.60 min **(G)** HPLC chromatogram of the 1 mg/mL chicoric acid standard showing a corresponding peak at RT ≈ 32.60 min **(H)** LC–MS spectrum of CAREOCI showing the characteristic molecular ion corresponding to chicoric acid (C_22_H_18_O_12_). **(I)** LC–MS spectrum of the 1 mg/mL chicoric acid standard confirming the corresponding molecular ion.

### 3.2 *In silico* screening

#### 3.2.1 Molecular docking insights of CAREOCI for beta cell preservation-related molecular targets

Molecular docking was performed to evaluate the binding affinity of chicoric acid with key proteins involved in β-cell function, oxidative stress, inflammation, and insulin signaling. Chicoric acid exhibited strong binding interactions with several targets, showing the highest docking scores with DYRK1A (−8.72), followed by mTOR (−7.7), RANKL (−7.6), TGF-β/TGF-βR (−7.61), NF-κB (−7.5), Exendin-4 (−7.4), and Cathepsin (−7.4Favorable docking scores were observed with Pdx-1 (−7.1), SMAD3 (−7.0), Mafa (−7.0), NFAT (−6.9), Elastase (−6.9), Pax-6 (−7.2), and IGF-1 (−7.33).Lower docking scores were recorded for Nkx6.1 (−5.6), FoxO1 (−5.8), Pax-4 (−6.2), Ngn3 (−5.6), NeuroD1 (−4.5), and CHOP (−5.5). Overall, the docking results suggest favorable interactions of chicoric acid with multiple molecular targets associated with β-cell survival, regeneration, and metabolic regulation. Based on the *in silico* analysis, DYRK1A, Pax-6, MAFA, Pdx-1, mTOR, NF-κB, Nkx6.1, and CHOP were selected as molecular targets. Based on the *in silico* analysis and available literature on β-cell preservation and regeneration (Ghasemi Gojani et al., 2024), eight genes (DYRK1A, PAX6, MAFA, PDX1, mTOR, NF-κB, NKX6.1, and CHOP) were selected for gene-expression analysis in the treatment groups (CAREOCI and chicoric acid) and control groups as molecular targets (Supplementary Table S2; Figure 2).These docking findings provide supportive computational evidence for the potential interaction of chicoric acid with the selected molecular targets.

**FIGURE 2:**
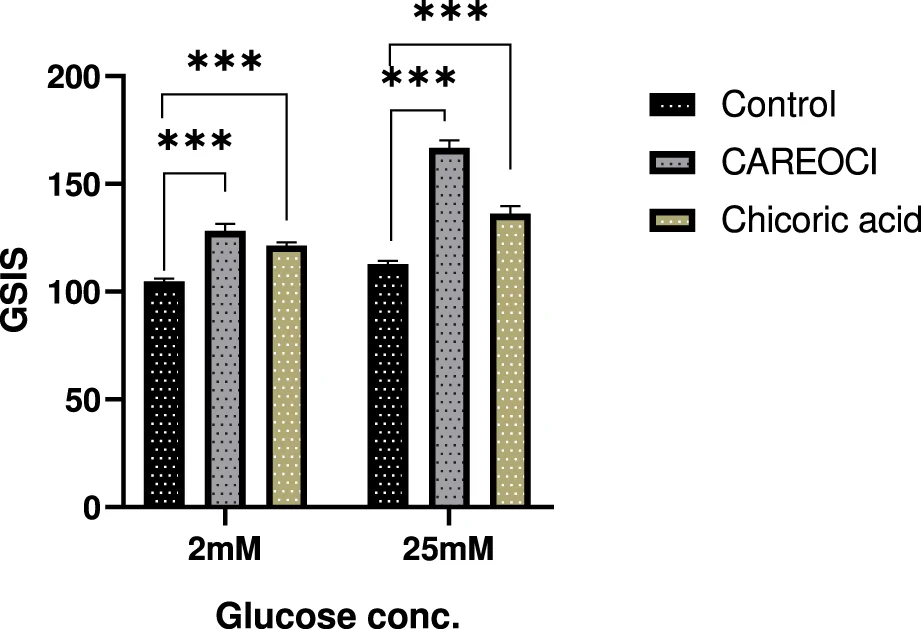
Effect of CAREOCI and chicoric acid on glucose-stimulated insulin secretion (GSIS) in MIN 6 β-cells under low (2 mM) and high (25 mM) glucose conditions. Data are presented as mean ± SD (n = 3); statistical significance was analyzed by one-way ANOVA followed by Tukey’s multiple comparison test (P < 0.05).

### 3.3 Effects of CAREOCI on β-cell function and survival under glucotoxic stress

#### 3.3.1 MTT cytotoxicity assay

CAREOCI treatment produced a concentration-dependent reduction in MIN6 cell viability, with viability decreasing from 95% at 10 μg/mL to 55% at 600 μg/mL and 10% at 1,000 μg/mL (Supplementary Figure S3). The concentration–response profile indicated an IC_50_ value of CAREOCI is 600 μg/mL.

#### 3.3.2 Glucose-stimulated insulin secretion

Glucose-stimulated insulin secretion (GSIS) was evaluated at 2 and 25 mM glucose. Insulin secretion was relatively lower at 2 mM glucose and increased following stimulation with 25 mM glucose across the experimental groups. At 2 mM glucose, GSIS was 104.71 ± 1.41, 128.21 ± 3.14, and 121.49 ± 1.40 for the control, CAREOCI, and chicoric acid groups, respectively. At 25 mM glucose, GSIS increased to 112.74 ± 1.52, 166.67 ± 3.67, and 136.30 ± 3.52, respectively (n = 3; Figure 2). Two-way ANOVA with Tukey’s multiple-comparison test showed significant differences among the treatment groups (p < 0.0001).

### 3.4 CAREOCI restores redox homeostasis and suppresses inflammation to protect MIN6 β-cells against glucotoxic stress

To investigate the protective effects of CAREOCI against glucose-induced oxidative stress and inflammation, intracellular antioxidant defence markers, lipid peroxidation, nitric oxide production, total antioxidant status, Trolox equivalent antioxidant capacity (TEAC), and pro-inflammatory cytokines were evaluated in glucotoxicity-induced MIN 6 β-cells following treatment.

CAREOCI significantly enhanced the endogenous antioxidant defense system, as evidenced by a marked increase in superoxide dismutase (SOD) activity to 14.13 ± 0.98 U/mg protein compared with both the normal-glucose Control 1 group (7.63 ± 0.56 U/mg protein; p < 0.0001) and the Control 2 group (11.63 ± 0.56 U/mg protein; p = 0.0061). In contrast, chicoric acid treatment resulted in an SOD activity of 10.61 ± 0.13 U/mg protein, which was significantly higher than Control 1 (p = 0.0018) but did not differ significantly from Control 2 (p = 0.1798). These findings indicate that CAREOCI produced a greater enhancement of SOD activity than chicoric acid alone, suggesting superior antioxidant efficacy of the whole extract. CAREOCI-treated cells exhibited an NO production rate of 10.60 ± 1.24 μM/min, compared with 7.40 ± 1.02 μM/min in the normal-glucose Control 1 group and 9.07 ± 0.60 μM/min in the Control 2 (25 mM glucose) group. CAREOCI did not significantly alter NO production compared with Control 2 (p = 0.1786), while chicoric acid treatment (9.00 ± 1.00 μM/min) also showed no significant difference from Control 2 (p = 0.9940). Notably, CAREOCI exhibited a significantly higher NO level than Control 1 (p = 0.0074), whereas chicoric acid did not (p = 0.1310). Overall, these findings suggest that the antioxidant effects of CAREOCI are not primarily associated with modulation of NO production under glucotoxic conditions. Lipid peroxidation, determined by malondialdehyde (MDA) levels, was 7.53 ± 0.78 μM/mg protein in the normal-glucose Control 1 group and decreased to 5.83 ± 0.48 μM/mg protein under glucotoxic conditions in the Control 2 (25 mM glucose) group. CAREOCI treatment resulted in an MDA level of 6.18 ± 0.22 μM/mg protein, which was not significantly different from either Control 2 (p = 0.5691) or Control 1 (p = 0.0899). In contrast, treatment with purified chicoric acid significantly increased MDA levels to 10.32 ± 0.56 μM/mg protein, which was significantly higher than both Control 2 (p < 0.0001) and Control 1 (p = 0.0036). This unexpected increase in MDA was not observed with CAREOCI treatment and therefore warrants further investigation. Further, the total antioxidant capacity (TAC) was 27.62 ± 2.09 μmol/mL in the normal-glucose Control 1 group and increased to 36.77 ± 1.17 μmol/mL under glucotoxic conditions in the Control 2 (25 mM glucose) group. CAREOCI treatment produced a marked elevation in TAC to 86.24 ± 0.58 μmol/mL, which was significantly higher than both Control 2 (p < 0.0001) and Control 1 (p < 0.0001). In contrast, chicoric acid treatment resulted in a TAC value of 35.33 ± 9.87 μmol/mL, showing no significant difference from either Control 2 (p = 0.9348) or Control 1 (p = 0.2989). The Trolox equivalent antioxidant capacity (TEAC) assay demonstrated a significant enhancement in non-enzymatic antioxidant capacity following CAREOCI treatment, with TEAC increasing to 0.316 ± 0.019 µM compared with 0.110 ± 0.051 µM in the Control 2 (25 mM glucose) group (p = 0.0004). CAREOCI also exhibited significantly higher TEAC than the normal-glucose Control 1 group (0.143 ± 0.014 µM; p = 0.0003). Chicoric acid treatment increased TEAC to 0.211 ± 0.006 µM, which was significantly higher than Control 2 (p = 0.0145) but did not differ significantly from Control 1 (0.143 ± 0.014 µM; p = 0.0733). Overall, CAREOCI produced a greater enhancement in TEAC than chicoric acid, indicating a stronger free-radical-scavenging capacity of the whole extract.

To further investigate the anti-inflammatory effects of CAREOCI, the levels of the pro-inflammatory cytokines interleukin-1 beta (IL-1β) and tumor necrosis factor-alpha (TNF-α) were assessed in glucotoxic MIN 6 cells. IL-1β levels were 76.97 ± 5.85 pg/mL in the normal-glucose Control 1 group and increased to 94.90 ± 0.04 pg/mL in the Control 2 (25 mM glucose) group. CAREOCI significantly reduced IL-1β levels to 86.90 ± 0.48 pg/mL compared with Control 2 (p < 0.0001). Chicoric acid also decreased IL-1β levels to 91.83 ± 0.02 pg/mL, representing a significant reduction compared with Control 2 (p = 0.0003), although the reduction was less pronounced than that observed with CAREOCI. Furthermore, IL-1β levels were significantly lower in the CAREOCI-treated group than in the chicoric acid-treated group (p < 0.0001).

A similar pattern was observed for TNF-α and it was 99.66 ± 0.10 pg/mL in the normal-glucose Control 1 group and increased to 124.90 ± 1.38 pg/mL under glucotoxic conditions. CAREOCI significantly suppressed TNF-α levels to 70.93 ± 0.52 pg/mL compared with Control 2 (p < 0.0001). Chicoric acid treatment also reduced TNF-α levels to 119.90 ± 1.87 pg/mL, producing a modest but statistically significant decrease compared with Control 2 (p = 0.0073). Notably, CAREOCI-treated cells exhibited significantly lower TNF-α levels than both Control 1 (p < 0.0001) and chicoric acid-treated cells, whereas chicoric acid-treated cells also differed significantly from Control 1 (p < 0.0001). These findings indicate that CAREOCI exerts a stronger anti-inflammatory effect than chicoric acid alone, particularly in suppressing glucotoxicity-associated cytokine responses.

Collectively, these findings demonstrate that CAREOCI exerts robust antioxidant and anti-inflammatory effects in glucotoxicity-induced MIN 6 β-cells. The extract markedly enhanced enzymatic and non-enzymatic antioxidant defenses, as evidenced by increased SOD, TAC, and TEAC levels, while NO production and lipid peroxidation were not significantly altered relative to the glucotoxic Control 2 group. Furthermore, CAREOCI markedly suppressed the pro-inflammatory cytokines IL-1β and TNF-α compared with Control 2, indicating attenuation of glucotoxicity-associated inflammatory responses. In comparison, isolated chicoric acid exhibited comparatively weaker antioxidant and anti-inflammatory effects. The superior activity of CAREOCI may therefore be attributable to the potential complementary effects and interactions among multiple bioactive phytoconstituents present in the extract (Figure 3).

**FIGURE 3:**
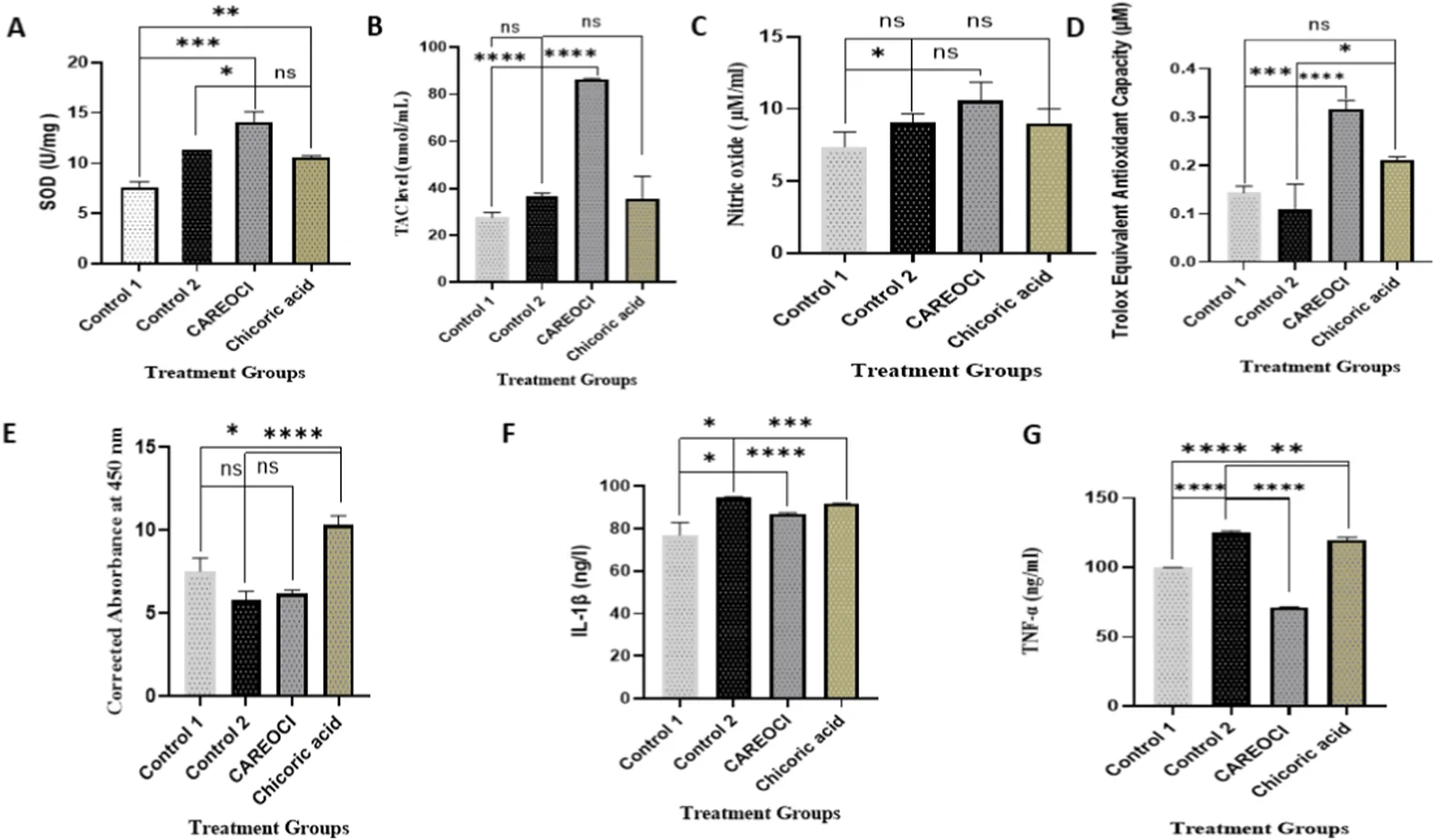
Effect of CAREOCI on oxidative stress and inflammatory markers in glucotoxicity-induced MIN 6 β-cells. **(A)** Superoxide dismutase (SOD) activity (U/mg protein), **(B)** total antioxidant capacity (TAC; µmol/mL), **(C)** nitric oxide (NO) levels (µM/min), **(D)** Trolox equivalent antioxidant capacity (TEAC; µM), **(E)** malondialdehyde (MDA) levels (corrected absorbance at 450 nm), **(F)** interleukin-1β (IL-1β; ng/mL), and **(G)** tumor necrosis factor-α (TNF-α; µg/mL) were assessed following 24 h of treatment in MIN 6 β-cells. Control 1 represents cells maintained under normal-glucose conditions, whereas Control 2 represents glucotoxicity induced by 25 mM glucose. Compared with the normal-glucose Control 1, glucotoxic conditions altered the oxidative stress and inflammatory parameters. CAREOCI treatment significantly enhanced SOD activity, TAC, and TEAC and markedly reduced IL-1β and TNF-α levels compared with Control 2. CAREOCI did not significantly alter NO or MDA levels compared with Control 2. In comparison, chicoric acid significantly increased TEAC and reduced IL-1β and TNF-α levels, but did not significantly affect SOD activity, TAC, or NO levels; notably, chicoric acid significantly increased MDA levels compared with Control 2. Data are presented as mean ± SD from three independent experiments (n = 3). Statistical significance was determined using one-way ANOVA followed by Tukey’s multiple-comparisons test. *p < 0.05, **p < 0.01, ***p < 0.001, ****p < 0.0001; ns, not significant.

Intracellular reactive oxygen species (ROS) generation was assessed using DCFH-DA staining followed by flow cytometric analysis to evaluate the effect of CAREOCI on oxidative stress in MIN6 β-cells (Figure 4). The normal-glucose Control 2 group exhibited 93.50% ± 0.69% ROS-positive cells, whereas the 25 mM glucose Control 1 group showed 90.26% ± 9.45% ROS-positive cells. The difference between Control 1 and Control 2 was not statistically significant, indicating that the glucotoxic condition did not significantly alter the percentage of ROS-positive cells under the present experimental conditions. CAREOCI treatment markedly reduced ROS-positive cells to 60.13% ± 2.95%, which was significantly lower than both Control 2 (p = 0.0006) and Control 1 (p = 0.0115). Chicoric acid treatment resulted in 81.15% ± 3.99% ROS-positive cells, showing a significant reduction compared with Control 2 (p = 0.0253), and Control 1 (p = 0.3212). Thus, CAREOCI produced a more pronounced reduction in ROS-positive cells than isolated chicoric acid, particularly relative to the glucotoxic Control 1 group. These findings indicate that CAREOCI possesses a stronger ROS-reducing effect under the experimental conditions, which may reflect the complementary or additive antioxidant activities of multiple phytoconstituents present in the extract (Figure 4).

**FIGURE 4:**
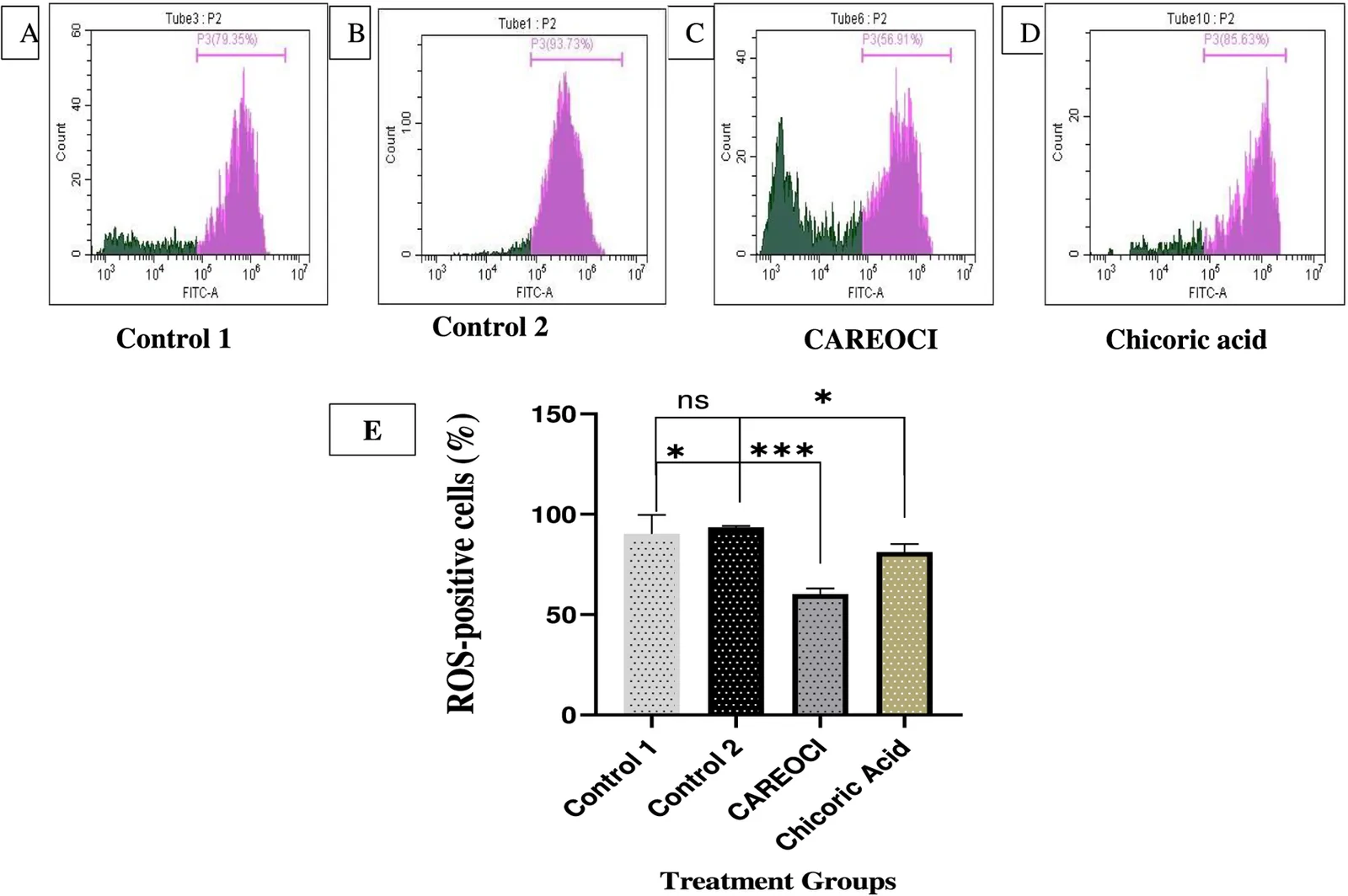
Effect of CAREOCI on intracellular reactive oxygen species (ROS) generation in glucotoxicity-induced MIN 6 β-cells. Intracellular ROS generation was assessed using DCFH-DA staining followed by flow cytometric analysis. **(A)** Representative flow cytometric histogram of Control 1 cells maintained under normal-glucose conditions. **(B)** Representative histogram of glucotoxic Control 2 cells exposed to 25 mM glucose. **(C)** Representative histogram of CAREOCI-treated cells. **(D)** Representative histogram of chicoric acid-treated cells. **(E)** Quantitative analysis of ROS-positive cells expressed as the percentage of cells within the P3 gate. CAREOCI treatment significantly reduced the proportion of ROS-positive cells compared with both Control 1 and glucotoxic Control 2, whereas chicoric acid did not significantly alter ROS-positive cells compared with Control 2. Data are presented as mean ± SD from three independent experiments (n = 3). Statistical analysis was performed using one-way ANOVA followed by Tukey’s multiple-comparisons test. *p < 0.05, **p < 0.01, ***p < 0.001; ns, not significant.

### 3.5 Effect of CAREOCI on β-cell function, survival, and proliferation-associated genes

Relative mRNA expression of β-cell identity, functional, inflammatory, and stress-associated genes were analyzed after 24 h of treatment with CAREOCI and purified chicoric acid under normal-glucose and glucotoxic conditions, with normalization to β-actin (Figure 5). Under normal-glucose conditions (Control 1), CAREOCI upregulated the β-cell identity- and function-associated genes MAFA, PAX6, and PDX1, while DYRK1A, NF-κB, mTOR, and CHOP were comparatively downregulated. However, none of these changes were statistically significant (p > 0.05). Under glucotoxic conditions, compared with Control 2, CAREOCI upregulated MAFA, PDX1, PAX6, and NKX6.1, indicating preservation of β-cell identity and functional characteristics. In contrast, DYRK1A, NF-κB, mTOR, and CHOP were significantly downregulated (p = 0.0211, 0.0322, 0.0211, and 0.0307, respectively), suggesting attenuation of pathways associated with cellular stress, inflammation, and ER-stress responses.

**FIGURE 5:**
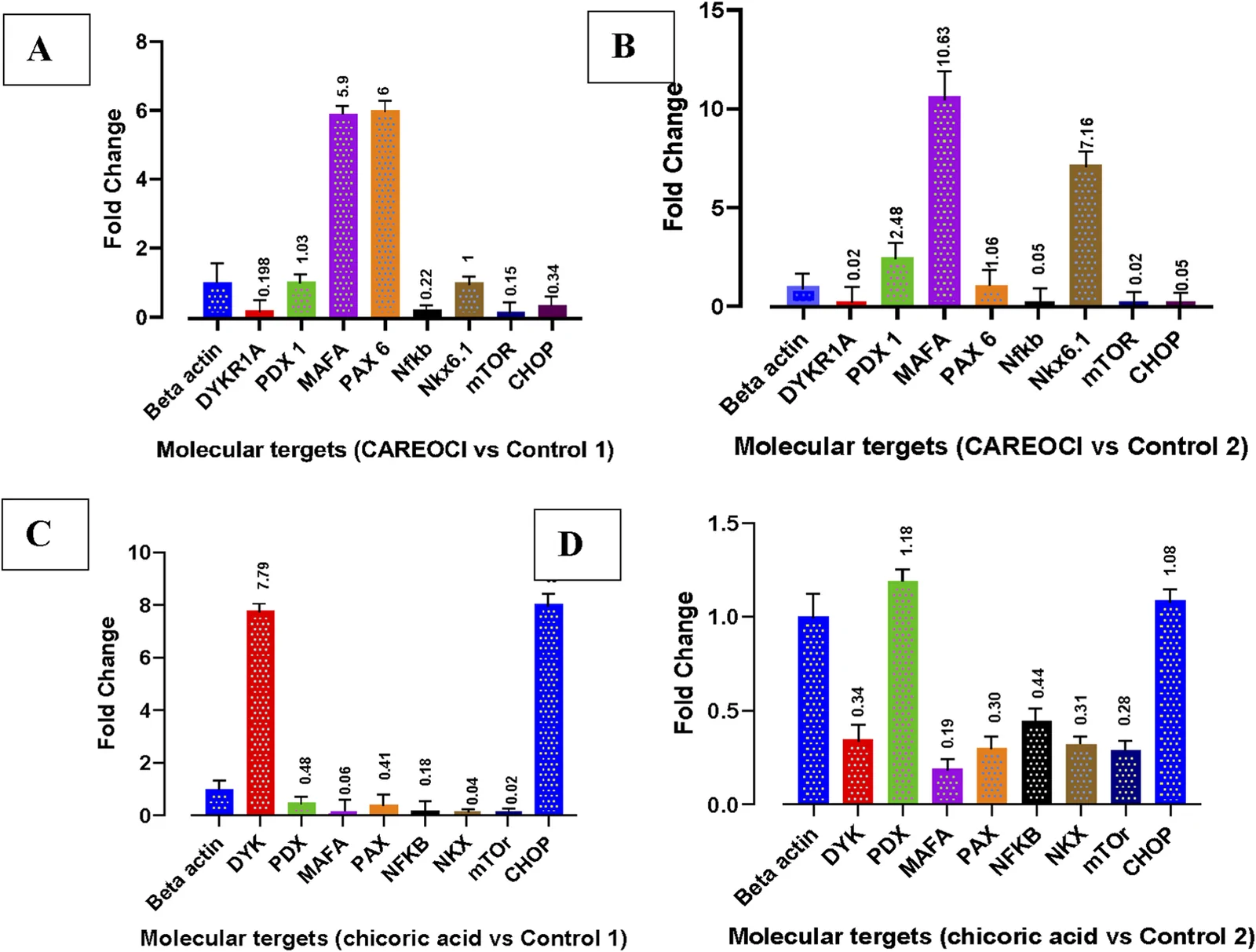
Relative mRNA expression of β-cell identity, functional, inflammatory, and stress-associated genes. Relative mRNA expression of DYRK1A, PDX1, MAFA, PAX6, NF-κB, NKX6.1, mTOR, and CHOP in MIN6 cells after 24 h of treatment with CAREOCI and chicoric acid. The expression of each target gene was normalized to the corresponding β-actin expression and presented relative to Control 1 (normal-glucose control) and Control 2 (glucotoxic control). Data are expressed as relative fold change compared with Control 1 **(A,C)** and control 2 **(B,D)**. Relative expression values > 1 indicate upregulation, whereas values < 1 indicate downregulation, relative to respective control groups.

Purified chicoric acid exhibited a comparatively distinct transcriptional response. Under normal-glucose conditions, DYRK1A and CHOP were upregulated, whereas β-cell identity-associated genes showed less pronounced induction. Under glucotoxic conditions, chicoric acid modulated PDX1, PAX6, NKX6.1, NF-κB, mTOR, and CHOP, while MAFA remained comparatively downregulated. Overall, CAREOCI produced a more favorable transcriptional profile, characterized by upregulation of β-cell identity and function-associated genes and downregulation of inflammatory and stress-associated genes under glucotoxic conditions. This pattern suggests that CAREOCI may support β-cell preservation and functional maintenance more effectively than purified chicoric acid (Figure 5).

### 3.6 Effect of CAREOCI on cell cycle progression

Cell-cycle distribution of MIN6 cells was evaluated by propidium iodide staining followed by flow-cytometric analysis (Figure 6). Most cells were distributed in the G0/G1 phase, with fewer cells in the S and G2/M phases. The G0/G1 populations were 63.34% ± 1.17%, 65.95% ± 2.46%, 58.41% ± 1.24%, and 67.90% ± 0.37% in Control 1, Control 2, CAREOCI, and chicoric acid groups, respectively. The corresponding S-phase populations were 10.67% ± 0.45%, 13.22% ± 0.82%, 19.75% ± 2.16%, and 14.56% ± 1.10%, while G2/M populations were 6.30% ± 0.36%, 7.21% ± 1.38%, 6.22% ± 5.15%, and 5.83% ± 0.91%,in Control 1, Control 2, CAREOCI, and chicoric acid groups, respectively (n = 3).

**FIGURE 6:**
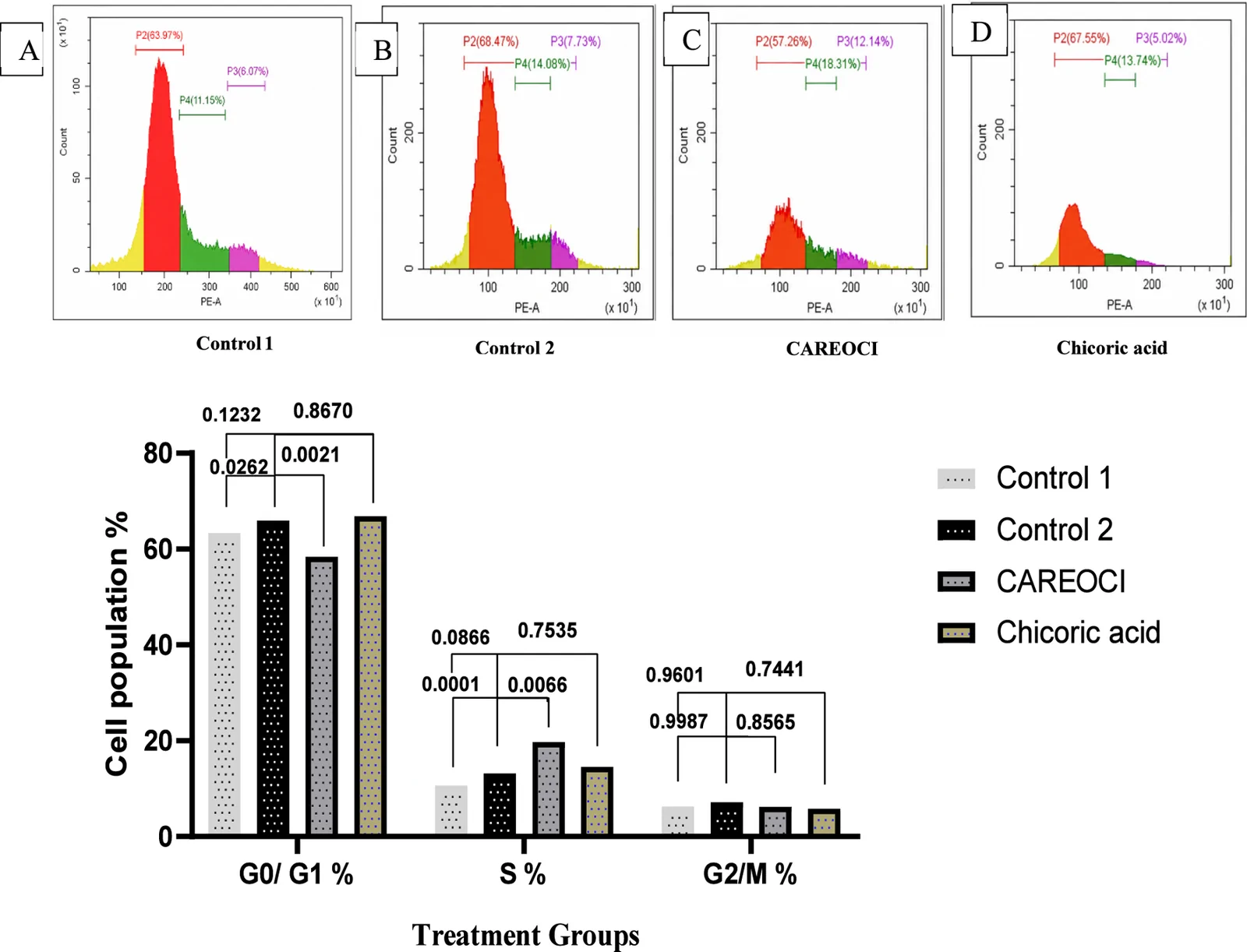
Effect of CAREOCI on cell-cycle distribution in MIN 6 β-cells. Cell-cycle distribution was assessed by propidium iodide (PI) staining followed by flow cytometric analysis. **(A)** Representative cell-cycle histogram of Control 1 cells maintained under normal-glucose conditions. **(B)** Representative histogram of glucotoxic Control 2 cells exposed to 25 mM glucose. **(C)** Representative histogram of CAREOCI-treated cells. **(D)** Representative histogram of chicoric acid-treated cells. **(E)** Quantitative analysis of the percentage of cells distributed across the G0/G1, S, and G2/M phases of the cell cycle. The effects of glucotoxicity and treatment on cell-cycle distribution were evaluated by comparing the respective treatment groups with the control conditions. Data are presented as mean ± SD from three independent experiments (n = 3). Statistical analysis was performed using two-way ANOVA. Statistical significance is indicated by the p-values shown in the figure.

CAREOCI significantly reduced the G0/G1 population and increased the S-phase population compared with Control 2 (p = 0.0262 and p = 0.006, respectively), whereas no significant differences were observed between Control 1 and CAREOCI for G0/G1 (p = 0.8670) or S phase (p = 0.0866). Chicoric acid significantly increased the G0/G1 population compared with Control 1 (p = 0.0021). No significant differences were observed in the G2/M phase among groups (p > 0.05). Overall, CAREOCI promoted G0/G1-to-S phase progression under glucotoxic conditions, suggesting increased cell-cycle activity.

### 3.7 Effect of CAREOCI on apoptosis

#### 3.7.1 Apoptosis assessment by annexin V FITC/PI assay

The anti-apoptotic effect of CAREOCI was evaluated using the Annexin V–FITC/PI assay (Figure 7). The percentage of apoptotic cells was 2.53% ± 0.25%, 51.98% ± 6.26%, 30.14% ± 6.06%, and 46.97% ± 14.11% in Control 1 (normal glucose), Control 2 (glucotoxic), CAREOCI, and chicoric acid groups, respectively (n = 3). Compared with Control 1, the control 2 group showed a marked increase in the apoptotic population, indicating substantial glucotoxicity-associated apoptotic stress. CAREOCI significantly reduced apoptosis compared with Control 2, decreasing the apoptotic population from 51.98% ± 6.26% to 30.14% ± 6.06% (p = 0.0484), corresponding to an approximately 42.0% reduction based on the group means. In contrast, chicoric acid treatment resulted in 46.97% ± 14.11% apoptotic cells and did not significantly differ from Control 2 (p = 0.8786). Overall, these findings indicate that CAREOCI significantly attenuated the glucotoxicity-associated increase in apoptosis, whereas purified chicoric acid did not produce a significant anti-apoptotic effect under the experimental conditions.

**FIGURE 7:**
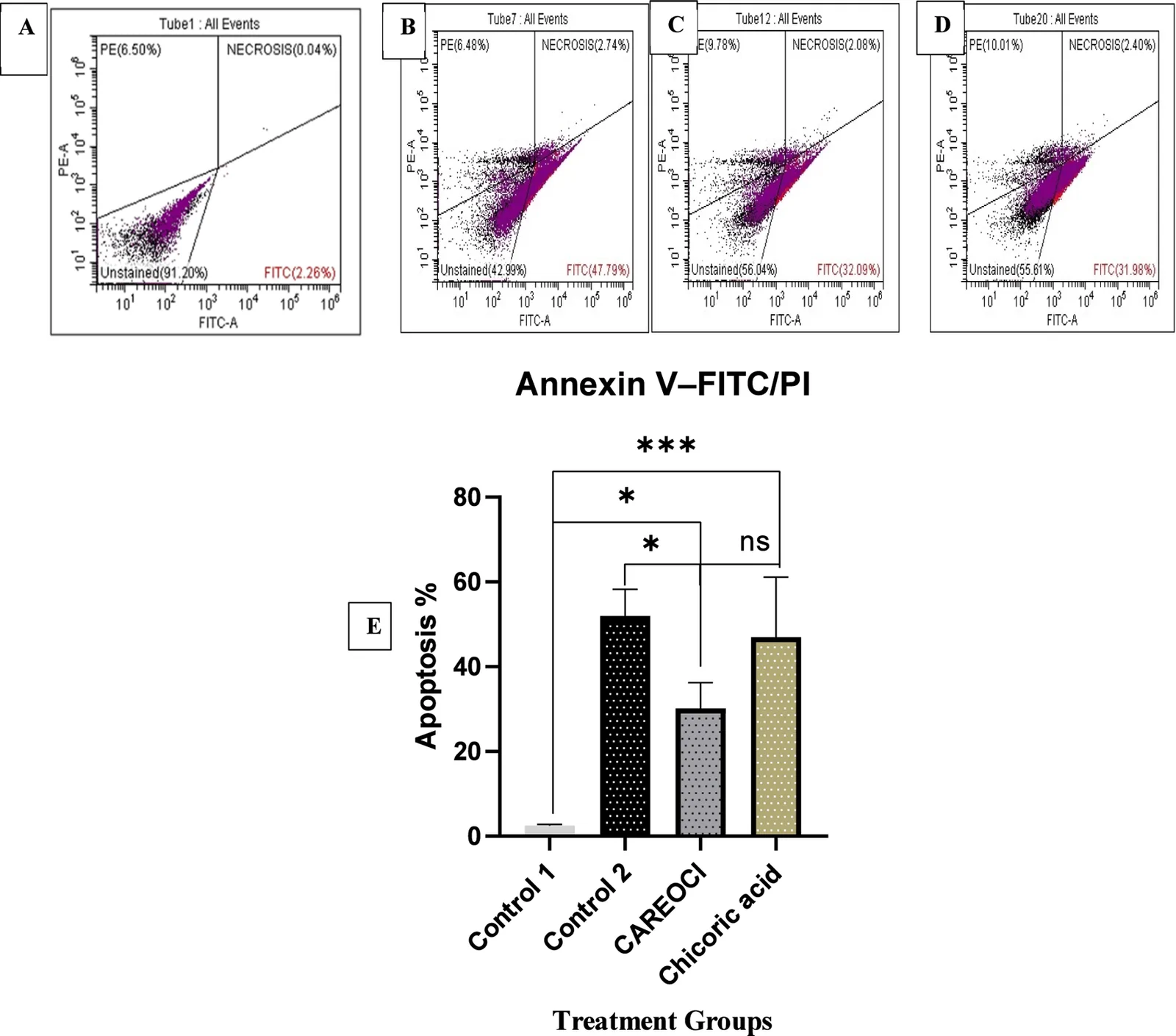
Effect of CAREOCI and chicoric acid on apoptosis in glucotoxicity-induced MIN 6 β-cells. Apoptosis was assessed by Annexin V–FITC/PI staining followed by flow cytometric analysis. **(A)** Representative Annexin V–FITC/PI dot plot of Control 1 cells maintained under normal-glucose conditions. **(B)** Representative dot plot of glucotoxic Control 2 cells exposed to 25 mM glucose. **(C)** Representative dot plot of CAREOCI-treated cells. **(D)** Representative dot plot of chicoric acid-treated cells. **(E)** Quantitative analysis of apoptotic cell populations expressed as a percentage relative to the control group. CAREOCI treatment significantly reduced apoptosis compared with the glucotoxic Control 2 group, whereas chicoric acid did not significantly alter apoptosis compared with Control 2. Data are presented as mean ± SD from three independent experiments (n = 3). Statistical analysis was performed using one-way ANOVA followed by Tukey’s multiple-comparisons test. *p < 0.05; ns, not significant.

#### 3.7.2 CAREOCI attenuates caspase-3/7 activity in MIN 6 cells

Caspase-3/7 activity was assessed in MIN 6 cells following treatment. The activity levels were 0.89 ± 0.02-fold, 1.00 ± 0.00-fold, 0.59 ± 0.09-fold, and 0.92 ± 0.14-fold in Control 1 (normal glucose), Control 2 (25 mM glucose), CAREOCI, and chicoric acid groups, respectively (n = 3). CAREOCI significantly reduced caspase-3/7 activity compared with Control 2 (p < 0.01), corresponding to an approximately 41% reduction. Chicoric acid produced a modest reduction (0.92 ± 0.14-fold) compared with Control 2, which was not statistically significant (p > 0.05). CAREOCI showed significantly lower caspase-3/7 activity than Control 1 (p = 0.0079), whereas chicoric acid did not differ significantly from Control 1 (p = 0.9286). These findings indicate that CAREOCI markedly attenuated glucotoxicity-associated caspase-3/7 activity (Figure 8).

**FIGURE 8:**
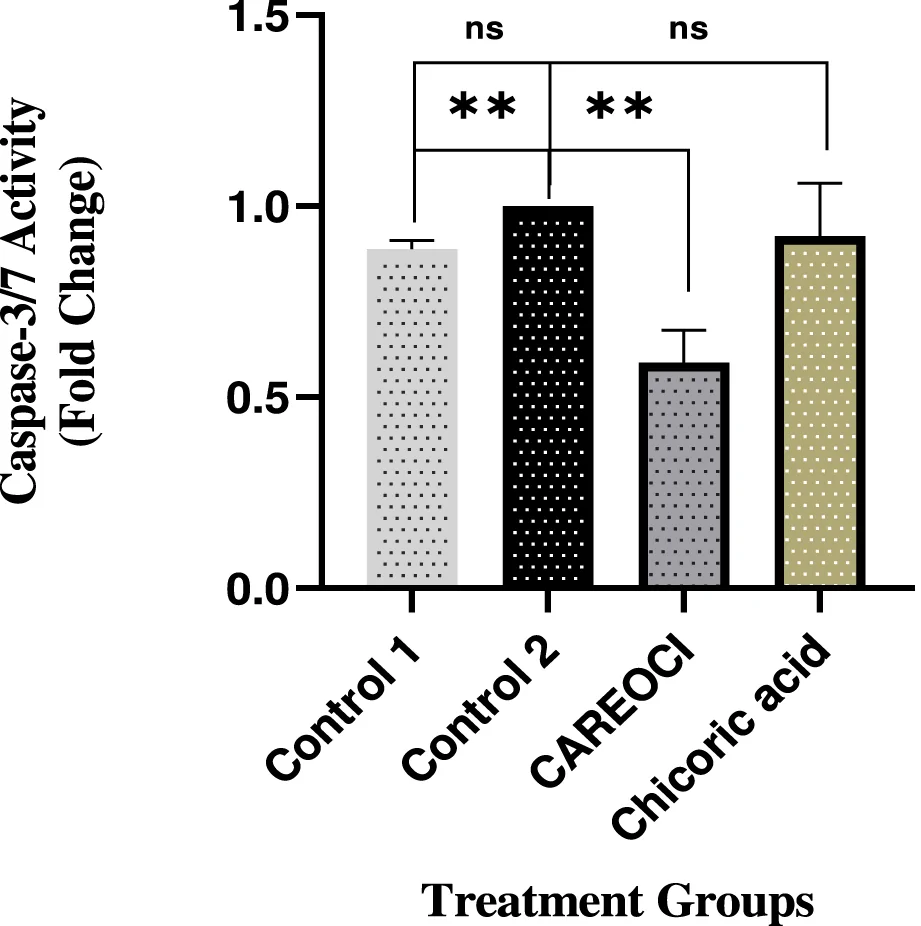
Effect of CAREOCI and chicoric acid on caspase-3/7 activity in MIN 6 β-cells. Caspase-3/7 activity was quantified using a luminescence-based assay and expressed as fold change relative to the glucotoxic Control 2 group (25 mM glucose). Control 1 represents cells maintained under normal-glucose conditions, whereas Control 2 represents cells exposed to 25 mM glucose. Compared with Control 2, CAREOCI treatment significantly reduced caspase-3/7 activity (**p < 0.01), whereas chicoric acid produced a non-significant reduction. Control 1 also exhibited significantly lower caspase-3/7 activity compared with Control 2 (**p < 0.01). Data are presented as mean ± SD from three independent experiments (n = 3). Statistical significance was determined using one-way ANOVA followed by Tukey’s multiple-comparisons test. **p < 0.01; ns, not significant.

#### 3.7.3 DAPI staining of nuclear morphology

DAPI staining was performed to assess nuclear morphology in MIN 6 cells in control and treatment groups. Control 1 (normal glucose) cells exhibited predominantly round-to-oval nuclei with intact nuclear architecture and homogeneous chromatin distribution. Control 2 (glucotoxic) cells showed increased nuclear condensation and irregular morphology, consistent with glucotoxicity-associated nuclear injury. Treatment with CAREOCI and chicoric acid preserved relatively intact nuclear morphology, with reduced chromatin condensation and fragmentation. The protective effect appeared more pronounced in the CAREOCI-treated group. These findings qualitatively support the cytoprotective and anti-apoptotic effects of CAREOCI and chicoric acid under glucotoxic conditions. The greater morphological preservation observed with CAREOCI may reflect potential complementary effects of its multiple phytoconstituents (Figure 9).

**FIGURE 9:**
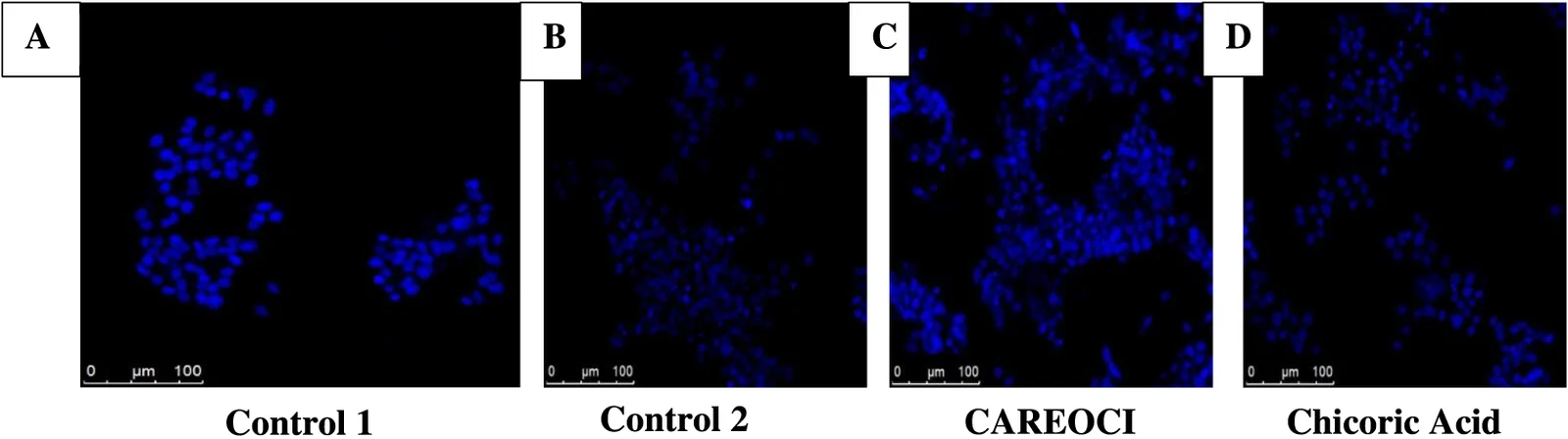
Representative confocal images of DAPI-stained MIN 6 β-cells following glucotoxicity and treatment. Representative confocal fluorescence images of DAPI-stained MIN 6 β-cells under different experimental conditions. **(A)** Control 1 cells maintained under normal-glucose conditions. **(B)** Glucotoxic Control 2 cells exposed to 25 mM glucose. **(C)** CAREOCI-treated cells. **(D)** Chicoric acid-treated cells. Compared with Control 1, Control 2 cells exposed to 25 mM glucose exhibited condensed, fragmented, and irregular nuclei, indicative of glucotoxicity-associated cellular injury. Treatment with CAREOCI and chicoric acid preserved nuclear morphology, reduced chromatin condensation and fragmentation, and maintained nuclear integrity. Blue fluorescence represents cell nuclei. Scale bar = 100 µm.

### 3.8 CAREOCI preserves mitochondrial membrane potential in MIN 6 cells

Mitochondrial membrane potential (ΔΨm) was evaluated using Rhodamine 123 (Rh123) staining in MIN 6 cells exposed to glucotoxic conditions (25 mM glucose for 24 h), followed by treatment with CAREOCI or purified chicoric acid for an additional 24 h. Control 2 exhibited altered green fluorescence compared with Control 1, indicating glucotoxicity-associated mitochondrial dysfunction. CAREOCI treatment markedly increased Rh123 fluorescence compared with Control 2, while chicoric acid also showed increased fluorescence, although with greater variability. Quantitative analysis showed relative Rh123 fluorescence of 62.56% ± 5.51%, 38.18% ± 11.47%, and 66.03% ± 8.22%, and 57.45% ± 23.00% in Control 1, Control 2, CAREOCI, and chicoric acid groups, respectively. CAREOCI significantly increased fluorescence compared with Control 2 (p = 0.0259), whereas chicoric acid did not (p = 0.1618). These findings indicate that CAREOCI significantly increased Rh123 fluorescence under glucotoxic conditions, supporting its potential protective effect against glucotoxicity-associated mitochondrial dysfunction (Figure 10).

**FIGURE 10:**
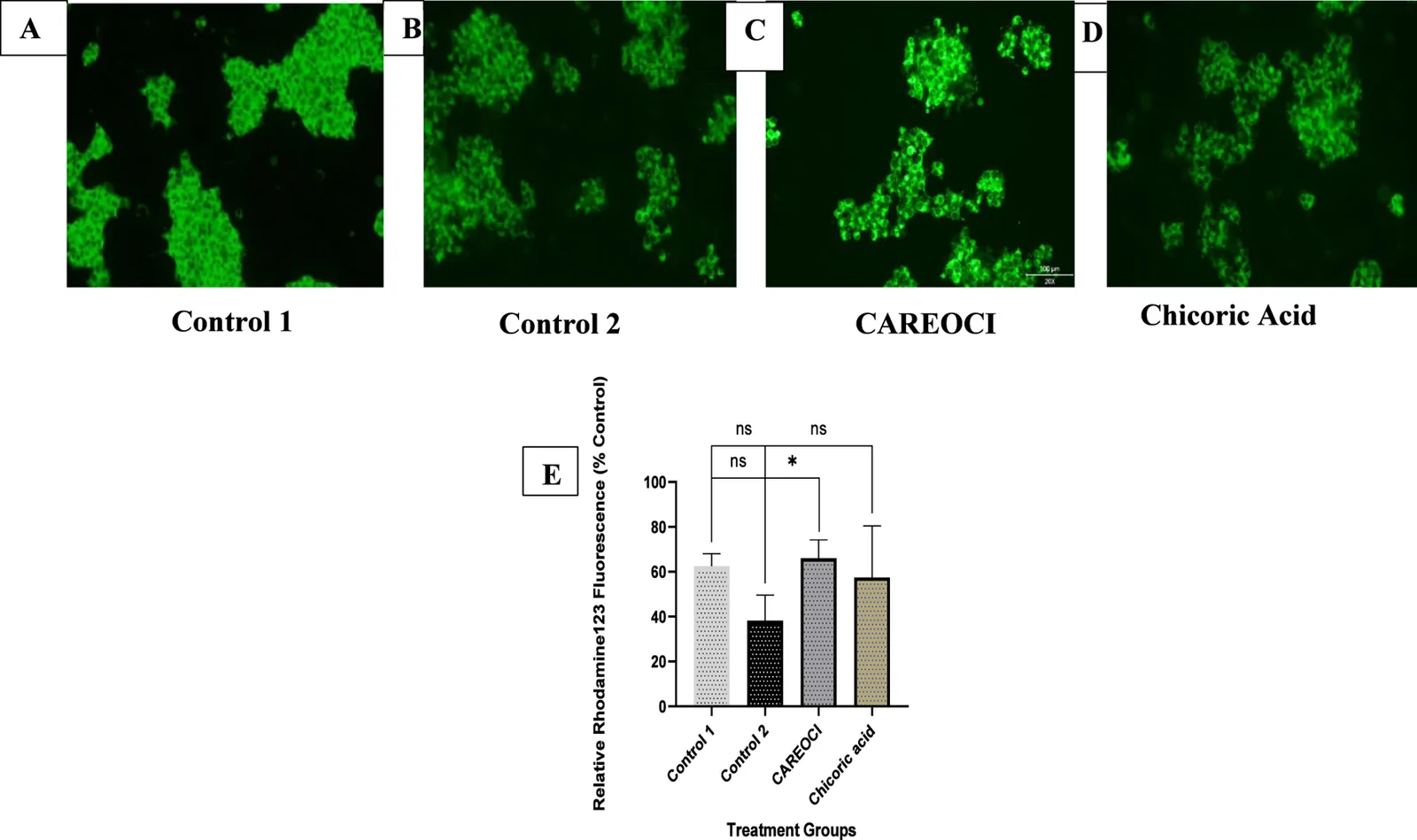
Effect of CAREOCI and chicoric acid on mitochondrial membrane potential (ΔΨm) in MIN 6 β-cells assessed by Rhodamine 123 staining. Mitochondrial membrane potential was evaluated using Rhodamine 123 fluorescence. **(A)** Representative fluorescence micrograph of Control 1 cells maintained under normal-glucose conditions. **(B)** Representative fluorescence micrograph of glucotoxic Control 2 cells exposed to 25 mM glucose. **(C)** Representative fluorescence micrograph of CAREOCI-treated cells. **(D)** Representative fluorescence micrograph of chicoric acid-treated cells. Green fluorescence represents Rhodamine 123 accumulation within mitochondria and reflects mitochondrial membrane potential. **(E)** Quantitative analysis of relative Rhodamine 123 fluorescence intensity expressed as a percentage of the glucotoxic Control 2 group. Glucotoxicity significantly reduced mitochondrial membrane potential compared with Control 1, whereas CAREOCI treatment significantly increased Rhodamine 123 fluorescence compared with Control 2, indicating restoration of mitochondrial membrane potential. Chicoric acid treatment showed no significant difference compared with Control 2. Data are presented as mean ± SD (n = 5). Statistical significance was determined using one-way ANOVA followed by Tukey’s multiple-comparisons test. *p < 0.05, **p < 0.01, ***p < 0.001; ns, not significant. Images were acquired at ×20 magnification. Scale bar = 100 µm.

### 3.9 CAREOCI restores autophagy in pancreatic β-cells

Autophagy-associated acidic vesicular organelles (AVOs) were assessed by acridine orange (AO) staining in MIN 6 cells (Figure 11). The AO red/green fluorescence ratios were 0.349 ± 0.143, 0.705 ± 0.100, 1.265 ± 0.100, 1.592 ± 0.143, and 0.937 ± 0.036 in Control 1, Control 2, rapamycin, CAREOCI, and chicoric acid groups, respectively (n = 3). Compared with Control 2, rapamycin significantly increased the AO ratio (p = 0.0004), while CAREOCI produced a greater increase (p < 0.0001). CAREOCI also showed a significantly higher AO ratio than rapamycin (p = 0.0089). Chicoric acid produced a modest increase compared with Control 2, which was not statistically significant (p = 0.0578). Compared with Control 1, significant differences were observed for rapamycin (p < 0.0001), CAREOCI (p < 0.0001), and chicoric acid (p = 0.0005). Thus, CAREOCI produced the highest AVO-associated fluorescence under the present experimental conditions. These findings indicate increased autophagy-associated AVO formation; however, AO staining alone does not establish increased autophagic flux (Figure 11).

**FIGURE 11:**
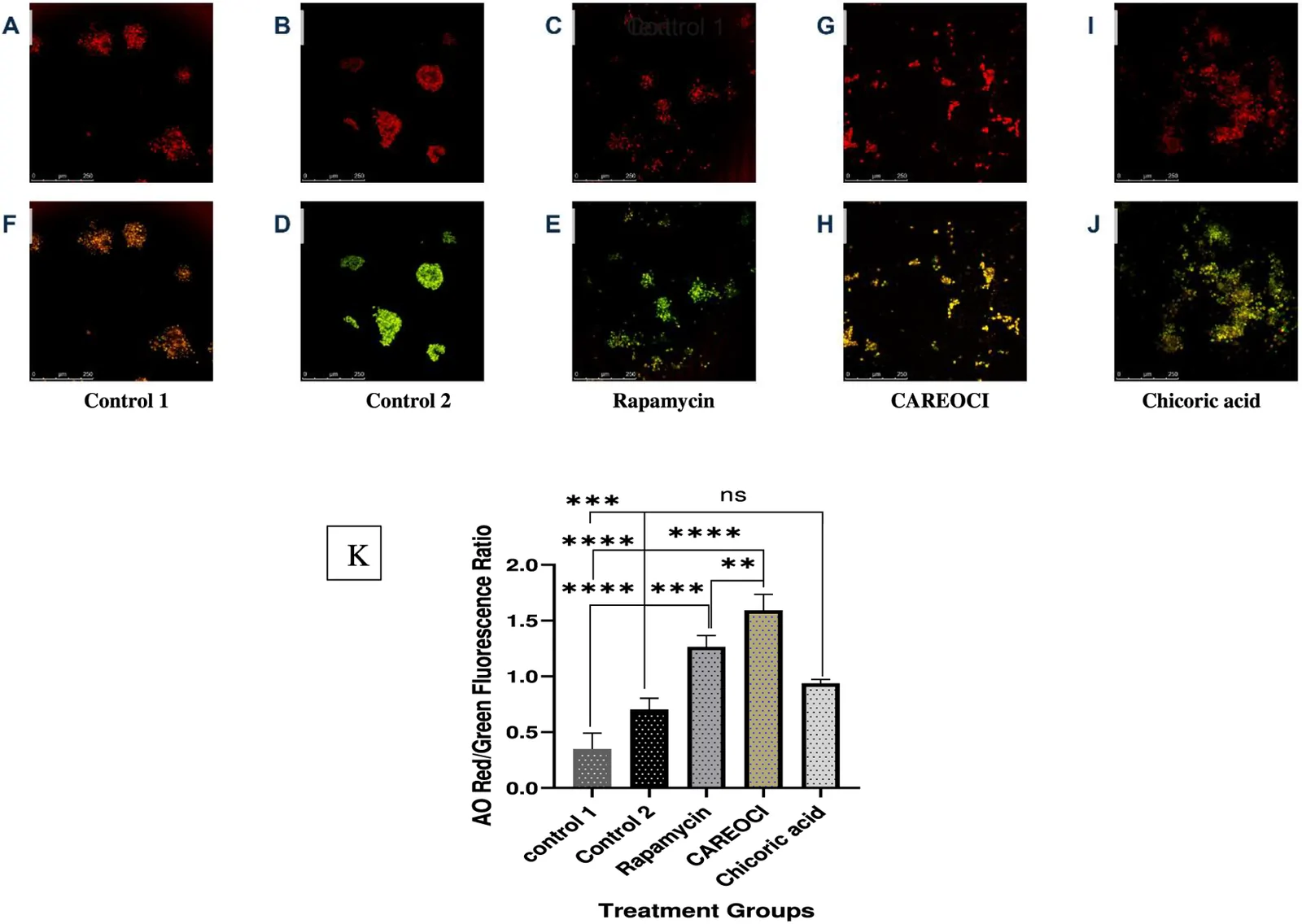
Acridine orange staining demonstrating CAREOCI-mediated induction of autophagy in glucotoxicity-induced MIN 6 β-cells. Representative confocal fluorescence images of MIN 6 β-cells stained with acridine orange under different experimental conditions. **(A,F)** Control 1 cells maintained under normal-glucose conditions, **(B,D)** glucotoxic Control 2 cells exposed to 25 mM glucose, **(C,E)** rapamycin-treated cells, **(G,H)** CAREOCI-treated cells, and **(I,J)** chicoric acid-treated cells. Red fluorescence represents the accumulation of acidic vesicular organelles (AVOs), indicative of increased autophagic activity, whereas the merged images demonstrate the intracellular localization of acidic vesicular compartments. **(K)** Quantitative analysis of the acridine orange red/green fluorescence ratio. Compared with Control 2, CAREOCI significantly increased the red/green fluorescence ratio, indicating enhanced autophagic activity under glucotoxic conditions. Rapamycin, used as a positive control for autophagy induction, also significantly increased the acridine orange red/green fluorescence ratio. Chicoric acid showed an increase in the red/green fluorescence ratio compared with Control 2. Data are presented as mean ± SD from three independent experiments (n = 3). Statistical significance was determined using one-way ANOVA followed by Dunnett’s multiple-comparisons test. ***p < 0.001 and ****p < 0.0001 *versus* CSontrol 2; **p < 0.01 *versus* the rapamycin-treated group; ns, not significant.

## 4 Discussion

The present study demonstrates that a standardized chicoric acid-rich extract of *C. intybus* (CAREOCI) effectively protects MIN6 pancreatic β-cells against glucose-induced glucotoxicity through the coordinated modulation of oxidative stress, inflammation, mitochondrial function, cell-cycle progression, apoptosis, autophagy, and β-cell preservation-associated pathways. Standardization of CAREOCI by HPTLC, HPLC, and LC-MS confirmed chicoric acid as the predominant phytochemical, together with hydroxycinnamic acids, alkaloids, phenolic derivatives, and quaternary amines, indicating that CAREOCI represents a chemically standardized phytopharmaceutical preparation rather than a single-compound preparation. Previous phytochemical investigations have identified chicoric acid as one of the principal bioactive constituents and quality markers of *C. intybus*, contributing to its antioxidant, anti-inflammatory, and antidiabetic properties (Lee and Scagel, 2013; Nasimi Doost Azgomi et al., 2021; Street et al., 2013). Furthermore, studies using *C. intybus*-derived preparations have demonstrated enhanced glucose uptake, stimulation of insulin secretion, and improved insulin sensitivity, supporting the antidiabetic potential of this plant (Azay-Milhau et al., 2013; Ferrare et al., 2018; Tousch et al., 2008).

Molecular docking identified DYRK1A as the most favourable molecular target among the selected targets for chicoric acid, exhibiting the strongest predicted binding affinity. DYRK1A negatively regulates NFAT-mediated β-cell proliferation, and its pharmacological inhibition has emerged as a promising strategy for stimulating β-cell regeneration and restoring β-cell mass (Karakose et al., 2018; Shirakawa and Kulkarni, 2016; Wang et al., 2015; Wang et al., 2019). Consistent with these computational findings, CAREOCI significantly downregulated DYRK1A expression while enhancing the expression of MAFA, PDX1, and PAX6, which are key transcription factors involved in maintaining β-cell identity, insulin biosynthesis, and functional maturation. Collectively, these findings suggest that CAREOCI modulates molecular pathways associated with β-cell preservation and potentially regenerative capacity, possibly involving DYRK1A/NFAT-associated signalling (Wang et al., 2015). However, the present study evaluated only at molecular-level expression changes and computational protein–ligand interactions rather than direct functional inhibition of DYRK1A. Therefore, the involvement of DYRK1A should be considered a potential mechanism rather than a definitively established molecular target of CAREOCI.

The gene-expression analysis further supported the role of CAREOCI in β-cell preservation under glucotoxic conditions. Compared with the glucotoxic Control 2 group, CAREOCI increased the expression of MAFA, PDX1, PAX6, and NKX6.1, indicating preservation of β-cell identity, maturation, and functional transcription. The importance of these transcription factors in maintaining β-cell identity and function is well established, and their dysregulation is associated with glucotoxicity-induced β-cell dysfunction (Schaffer et al., 2013; Taylor et al., 2013). The marked increases in MAFA and NKX6.1 are particularly relevant to the maintenance of mature β-cell function and insulin secretion, whereas PDX1 and PAX6 are important regulators of β-cell identity and endocrine differentiation (Schaffer et al., 2013; Zhu et al., 2017). Compared with Control 2, CAREOCI showed lower NF-κB and CHOP expression, indicating attenuation of glucotoxicity-associated inflammatory and ER-stress responses (Donath and Shoelson, 2011; Eizirik et al., 2008). Under Control 1 (normal-glucose) , CAREOCI similarly showed higher MAFA, PAX6, and PDX1 expression, supporting the maintenance of β-cell-associated transcriptional characteristics. In contrast, purified chicoric acid exhibited a distinct expression profile, particularly with higher DYRK1A and CHOP expression under both Control 1 and Control 2 groups. These findings suggest that CAREOCI may preserve β-cell identity and functional integrity while limiting glucotoxicity-associated inflammatory and cellular stress responses.

Glucose-stimulated insulin secretion (GSIS) is a critical indicator of β-cell functional competence and depends on coordinated glucose metabolism, mitochondrial activity, and stimulus–secretion coupling (Ashcroft and Rorsman, 2012; Henquin, 2009; Rorsman and Ashcroft, 2018). Under high-glucose conditions, CAREOCI significantly increased glucose-stimulated insulin secretion compared with the untreated high-glucose group, indicating improved glucose-responsive secretory activity and preservation of β-cell functional competence. Because insulin secretion is closely dependent on mitochondrial metabolism and intracellular redox balance, the restoration of GSIS further suggests that CAREOCI preserved stimulus–secretion coupling and β-cell function under glucotoxic stress (Ashcroft and Rorsman, 2012; Henquin, 2009; Rorsman and Ashcroft, 2018; Tousch et al., 2008). Previous studies have similarly reported beneficial effects of chicory-derived phenolics on glucose homeostasis and insulin responsiveness (Azay-Milhau et al., 2013; Ferrare et al., 2018).

Oxidative stress is a central mechanism underlying glucotoxic β-cell dysfunction because pancreatic β-cells possess relatively weak endogenous antioxidant defenses (Lenzen, 2008; Robertson, 2004). In agreement with this concept, CAREOCI significantly enhanced SOD activity, total antioxidant capacity, and TEAC while markedly reducing intracellular ROS generation, indicating an improvement in cellular redox homeostasis compared with glucotoxic Control 2. However, compared with normal-glucose Control 1, CAREOCI showed a comparatively lower antioxidant response, suggesting that although CAREOCI effectively attenuated glucotoxic oxidative stress, its effects did not completely restore the antioxidant status to that observed under normal-glucose conditions. Similar antioxidant properties of chicoric acid have previously been reported, including reduction of oxidative stress, enhancement of endogenous antioxidant defenses, and improvement of glucose metabolism (Ferrare et al., 2018; Tousch et al., 2008). The preservation of nitric oxide production, together with the absence of a significant increase in lipid peroxidation, further suggests that CAREOCI helped maintain cellular redox balance under glucotoxic conditions. These findings highlight the potential of CAREOCI to protect β-cell function and cellular integrity against glucotoxic stress.

An unexpected finding was observed following treatment with purified chicoric acid, which significantly increased MDA levels compared with both the normal-glucose Control 1 and glucotoxic Control 2 groups, indicating enhanced lipid peroxidation under the present experimental conditions. Although chicoric acid is generally reported to possess antioxidant and free-radical-scavenging properties, phenolic compounds can exhibit context-dependent redox effects depending on concentration, exposure conditions, cellular redox status, and interactions with transition metals (Leopoldini et al., 2011; Sakihama et al., 2002). Further studies employing a range of chicoric acid concentrations and complementary markers of oxidative stress and lipid peroxidation are warranted to elucidate the underlying mechanism and establish whether the observed increase in MDA reflects a concentration-dependent or context-specific response to purified chicoric acid. Importantly, the substantially greater antioxidant efficacy of CAREOCI compared with isolated chicoric acid supports the hypothesis that potential complementary interactions among multiple phytoconstituents contribute to the overall biological activity of the standardized extract.

Inflammation also plays a critical role in β-cell dysfunction, with chronic glucotoxicity inducing inflammatory responses through activation of NF-κB and cytokine-mediated apoptotic signalling (Donath and Shoelson, 2011; Eizirik et al., 2020; Hotamisligil, 2006). In the present study, glucotoxic Control 2 showed increased IL-1β and TNF-α levels compared with normal-glucose Control 1, confirming the induction of an inflammatory response under glucotoxic conditions. CAREOCI effectively reduced both IL-1β and TNF-α levels compared with Control 2 and Control 1, indicating attenuation of glucotoxicity-associated inflammation. In contrast, purified chicoric acid showed a comparatively weaker reduction in these inflammatory markers. Previous studies have demonstrated that chicory-derived phenolics exert protective effects against metabolic dysfunction associated with diabetes (Azay-Milhau et al., 2013; Ferrare et al., 2018).

CAREOCI increased the proportion of cells in the S phase. Normal-glucose Control 1 showed no significant effect on S-phase progression, whereas glucotoxic Control 2 represented the glucotoxic condition for evaluating the effect of CAREOCI. In contrast, purified chicoric acid did not significantly increase the proportion of S-phase cells compared with either Control 1 or Control 2 under the present experimental conditions. These findings suggest that molecular changes associated with β-cell preservation or regenerative pathways may precede detectable alterations in cell-cycle progression, particularly during short-term exposure. Longer treatment periods and direct proliferation assays may be required to determine whether these molecular changes translate into increased β-cell proliferation. Nevertheless, the concurrent suppression of DYRK1A and increased expression of MAFA, PDX1, and PAX6 following CAREOCI treatment suggest modulation of molecular pathways associated with β-cell functional maintenance and potentially proliferative capacity (Kumar et al., 2021).Importantly, the present findings do not establish that CAREOCI directly induces β-cell proliferation.

The present study showed the pronounced anti-apoptotic effect of CAREOCI. These observations indicate that CAREOCI effectively protects β-cells from glucotoxicity-induced programmed cell death compared with both Control 1 (normal-glucose) and Control 2 (glucotoxic) groups. Since progressive β-cell apoptosis is a major pathological feature of type 2 diabetes, inhibition of apoptotic signaling represents an important therapeutic objective (Butler et al., 2003; Cnop et al., 2017; Eizirik et al., 2020). In contrast, purified chicoric acid failed to provide comparable protection in comparison with both Control 1 and Control 2 groups. Further supporting the concept that potential complementary interactions among multiple phytochemicals present in CAREOCI contributes substantially to its cytoprotective efficacy.

Maintenance of mitochondrial integrity is essential for β-cell survival and glucose-stimulated insulin secretion. Rh123 staining demonstrated significant preservation of mitochondrial membrane potential following CAREOCI treatment, indicating improved mitochondrial function and bioenergetic capacity. Furthermore, acridine orange staining revealed a marked increase in acidic vesicular organelle formation, suggesting activation of protective autophagy. Similar observations have established autophagy as a fundamental adaptive response that preserves β-cell survival under metabolic stress (Marasco and Linnemann, 2018; Mizushima and Levine, 2010).

Overall, the present study demonstrates that CAREOCI exerts potent cytoprotective effects against glucotoxicity-induced injury in MIN 6 β-cells through multiple complementary mechanisms, including enhancement of antioxidant defenses, suppression of inflammatory cytokines, restoration of glucose-stimulated insulin secretion, preservation of mitochondrial function, inhibition of apoptosis, activation of autophagy, and modulation of β-cell regeneration-associated genes. The greater efficacy of CAREOCI compared with isolated chicoric acid, particularly the contrasting effects observed for lipid peroxidation, strongly suggests that potential complementary interactions among the diverse phytochemicals present in *C. intybus* contribute substantially to its biological activity. These findings provide mechanistic evidence supporting the therapeutic potential of standardized chicoric acid-rich *C. intybus* extract as a promising phytopharmaceutical for preserving β-cell function under glucotoxic conditions.

The present investigation was performed exclusively in an *in-vitro* MIN 6 cell model, further studies using primary human islets and diabetic animal models are required to validate these findings, elucidate the underlying molecular mechanisms, and determine their translational potential for diabetes therapy. A limitation of the present study is that autophagy was assessed using acridine orange staining, without additional autophagy-specific biomarkers, while molecular targets were evaluated only at the expression level without protein-level validation.

## 5 Conclusion

Chicoric acid-rich extract of *C. intybus* seeds (CAREOCI) protected pancreatic β-cells against glucotoxicity by enhancing insulin secretion and attenuating oxidative stress, inflammation, cell-cycle progression, apoptosis, and mitochondrial dysfunction. Furthermore, CAREOCI modulated key β-cell function-associated genes, including DYRK1A, MAFA, PDX1, and PAX6, supporting the maintenance of β-cell identity and function. CAREOCI showed greater cytoprotective and β-cell-preserving effects than purified chicoric acid, highlighting the potential contribution of its combined phytoconstituents.

## Data Availability

The raw data supporting of this article will be made available by the authors, without undue reservation.
