# Supplementary material for: Chicoric acid-rich extract of Cichorium intybus seeds (CAREOCI) preserves pancreatic β-cell function under glucotoxic stress through modulation of MAFA, DYRK1A, cell cycle, apoptosis, and mitochondrial dysfunction
Source: Front Pharmacol. 2026 Sep 30;17:1953632. doi: 10.3389/fphar.2026.1953632 (PMC13661457; doi:10.3389/fphar.2026.1953632)
Supplement: Supplementary file 1 [file DataSheet1.pdf]

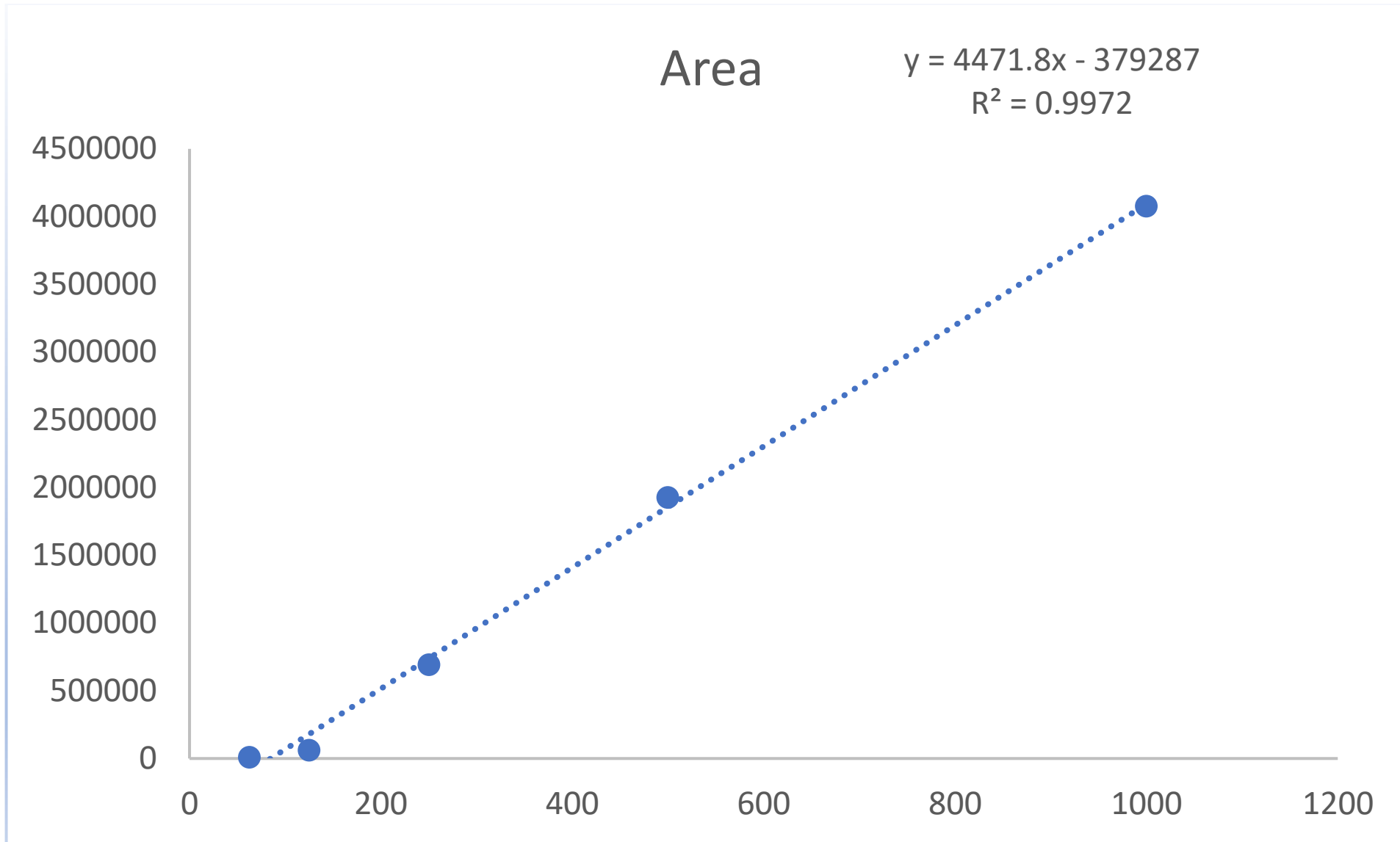

Supplementary Figure 1: HPLC Calibration Curve of Chicoric Acid *Calibration curve of chicoric acid. The calibration curve was generated using chicoric acid standards at concentrations of 62.5, 125, 250, 500, and 1000 µg/mL. The linear regression equation was* $y = 4471.8x - 379287$*, with a coefficient of determination (*$R^2 = 0.9972$*).*

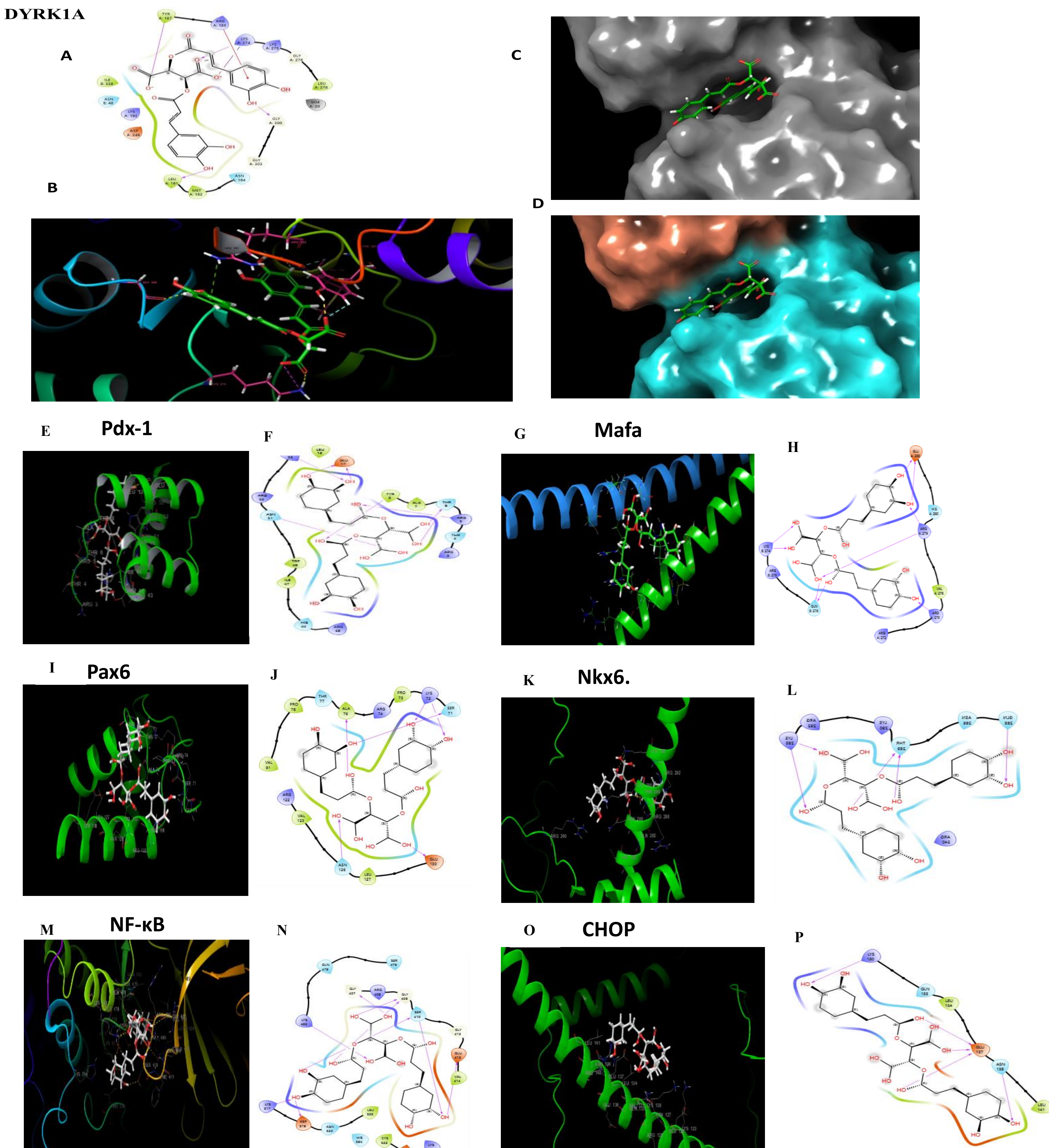
DYRK1A
A
B
C
D
E
Pdx-1
F
G
Mafa
H
I
Pax6
J
K
Nkx6.
L
M
NF-κB
N
O
CHOP
P

Q **mTOR** R

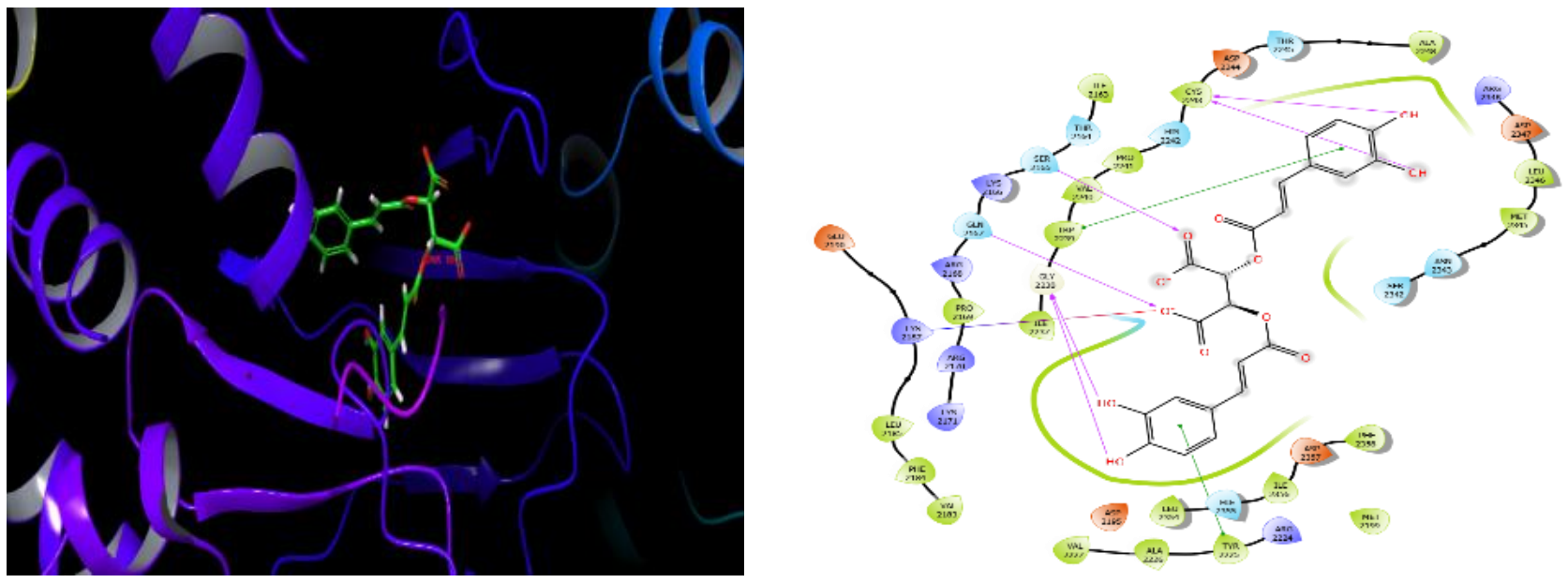

Supplementary Figure 2. *Molecular docking of chicoric acid with proteins involved in pancreatic β-cell regeneration and survival. (A–D) Binding interactions with DYRK1A; (E–P) docking poses and 2D interaction maps for PDX-1, MAFA, PAX6, NKX6.1, NF-κB, and CHOP; and (Q, R) 3D binding pose and 2D interaction map of mTOR. Protein–ligand interactions were visualized using Discovery Studio Visualizer.*

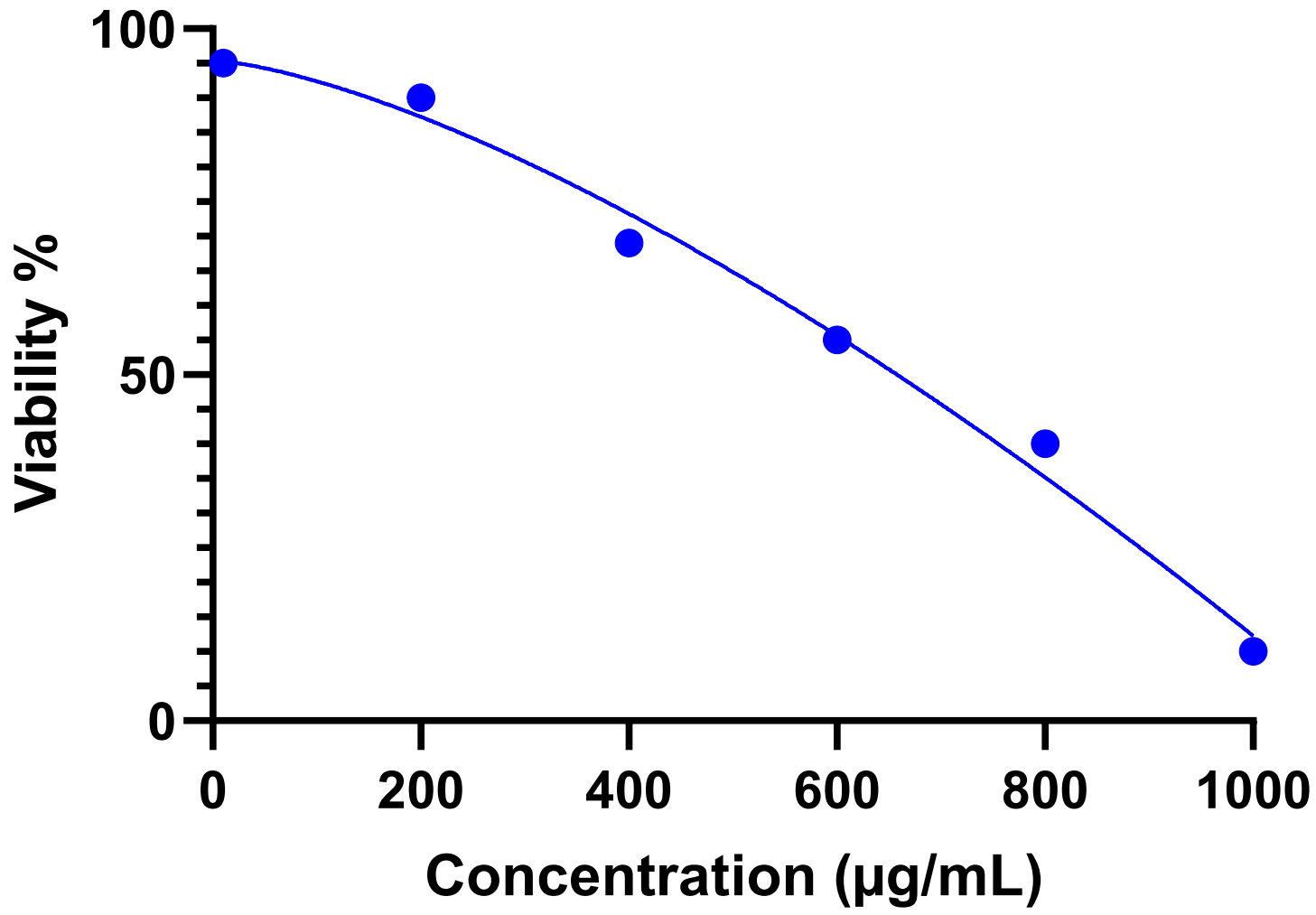

Supplementary ***Figure 3. Determination of the IC₅₀ of CAREOCI in MIN6 cells by MTT assay.*** *MIN6 cells were treated with increasing concentrations of CAREOCI (10, 200, 400, 600, 800, and 1000 µg/mL) for 24 h, and cell viability was assessed using the MTT assay. The IC₅₀ of CAREOCI was approximately 600 µg/mL, and this concentration was selected for subsequent experiments.*

Supplementary Table 1. Tentative identification of phytochemicals in the chicoric acid-rich aqueous reflux extract of *Cichorium intybus* (CAREOCI) by LC–ESI(+)-QTOF–MS analysis.

RT (min)	Observed m/z ([M+H]+)	Tentative Identification	Molecular Formula	Neutral MW (Da)	Compound Class	MSI Level
0.7160	104.1060	Choline	$C_5H_{13}NO$	103.17	Quaternary amine	MSI-2
0.8954	138.0532	Trigonelline	$C_7H_7NO_2$	137.12	Alkaloid	MSI-3
6.8251	475.3204	Chicoric acid	$C_{22}H_{18}O_{12}$	474.37	Hydroxycinnamic acid	MSI-2
12.6149	167.0687	Vanillic acid derivative	$C_8H_8O_4$	168.15	Phenolic acid	MSI-3
12.6149	191.1777	Quinic acid derivative	$C_7H_{12}O_6$	192.17	Organic acid	MSI-3
12.6149	297.0802	Caffeoylquinic acid derivative	—	—	Hydroxycinnamic acid	MSI-3
0.8954	462.3450	Flavonoid glycoside	—	—	Flavonoid	MSI-3
10.9063	464.3296	Flavonoid glycoside	—	—	Flavonoid	MSI-3
10.9063	453.3397	Phenolic acid derivative	—	—	Phenolic acid	MSI-3
6.8251	453.3388	Phenolic acid derivative	—	—	Phenolic acid	MSI-3
10.9063	348.2715	Unknown metabolite	—	—	Unknown	MSI-4
9.3048	340.2574	Coumarin glycoside (tentative)	—	—	Coumarin	MSI-3
12.6149	536.1606	Phenolic glycoside	—	—	Phenolic glycoside	MSI-3
0.8954	697.5155	Diglycosylated flavonoid	—	—	Flavonoid glycoside	MSI-4
9.3048	679.5054	Unknown glycosylated metabolite	—	—	Unknown	MSI-4
9.3048	701.4872	Unknown glycosylated metabolite	—	—	Unknown	MSI-4
17.4573–17.8706	551.4975–653.5821	Lipid-related metabolites (multiple ions)	—	—	Lipids/Glycerolipids	MSI-4

Supplementary Table 1 MSI identification levels follow the Metabolomics Standards Initiative (MSI): Level 1 = confirmed with authentic standard; Level 2 = probable structure based on accurate mass and/or MS/MS library match; Level 3 = tentative compound class or putative annotation; Level 4 = unknown metabolite.*

Supplementary Table 2 . Molecular docking scores of chicoric acid against β-cell regeneration- and diabetes-associated target proteins.

S. No.	Molecular target	PDB ID	Predicted docking score (kcal/mol)
1.	**DYRK1A**	**3ANQ**	**−8.72**
2.	**mTOR**	**8ERA**	**−7.70**
3.	**NF-κB**	**4G3D**	**−7.50**
4.	**PAX6**	**PAX6**	**−7.20**
5.	**PDX1**	**2H1K**	**−7.10**
6.	**MAFA**	**4EOT**	**−7.00**
7.	**CHOP/DDIT3**	**P35638**	**−5.50**
8.	**NKX6.1**	**P78426**	**−5.60**
9.	RANKL	3URF	−7.60
10.	TGF-β/TGF-βR	3KFD	−7.61
11.	Exendin-4	7LLL	−7.40
12.	Cathepsin G	1KYN	−7.40
13.	SMAD3	5XOC	−7.00
14.	NFAT	1OWR	−6.90
15.	Elastase	3Q76	−6.90
16.	IGF-1	1IMX	−7.33
17.	FOXO1	6QVW	−5.80
18.	PAX4	AF-O43316-F1	−6.20
19.	NGN3	AF-Q9Y422	−5.60
20.	NeuroD1	2QL2	−4.50

Supplementary Table 3. Primer sequences used for quantitative real-time PCR (qRT-PCR).

S. No.	Gene	Forward Primer (5′–3′)	Reverse Primer (5′–3′)
1	**β-Actin**	TTTCCAGCCTTCCTTCTTG	TGGCATAGAGGTCTTTACGG
2	**Dyrk1a**	TCAGTCTTCAGGCACCACCT	TGTTACTCGTTCCCGAGGAT
3	**Pdx1**	ATTCTTGAGGGCACGAGAGC	CTGGTCCGTATTGGAACGCT
4	**Mafa**	CAAGGAGGAGGTCATCCGAC	TCTCCAGAATGTGCCGCTG
5	**Pax6**	TAGCCCAGTATAAACGGGAGTG	CCAGGTTGCGAAGAACTCTG
6	**NF-κB**	GCTGCCAAAGAAGGACACGACA	GGCAGGCTATTGCTCATCACAG
7	**Nkx6.1**	CCCGGAGTGATGCAGAGTC	AGAACGTGGGTCTGGTGTGT
8	**mTOR**	CACCAGAATTGGCAGATTTGC	CTTGGACGCCATTTCCATGAC
9	**CHOP**	TTCACTACTCTTGACCCTGCGT	CACTGACCACTCTGTTTCCGTTTC
